# 14-3-3ε inhibits premature centriole disengagement by inhibiting the activity of Plk1 and separase

**DOI:** 10.1242/jcs.263808

**Published:** 2025-07-18

**Authors:** Monika A. Jaiswal, Akshay Karn, Aparna Das, Anisha Kumari, Shilu Tiwari, Sorab N. Dalal

**Affiliations:** ^1^ Cell and Tumour Biology, Advanced Centre for Treatment Research and Education in Cancer (ACTREC), Tata Memorial Centre, Kharghar Node, Navi Mumbai 410210, India; ^2^ Homi Bhabha National Institute, Training School Complex, Anushakti Nagar, Mumbai 400085, India

**Keywords:** 14-3-3ε, Centriole disengagement, Centrosome cycle, Plk1, Separase

## Abstract

The 14-3-3 protein family regulates several pathways in mammalian cells, including centrosome duplication. However, the precise mechanisms by which 14-3-3 paralogs regulate the centrosome cycle remain unclear. To identify the mechanisms by which 14-3-3ε regulates centrosome duplication, we altered two conserved acidic residues in the 14-3-3ε phospho-peptide-binding pocket that regulate complex formation and dissociation with the associated ligands, D127 and E134, to alanine. Altering these residues to alanine led to opposing effects on centrosome duplication; the D127A mutant inhibited centrosome duplication, whereas cells expressing the E134A mutant showed the presence of supernumerary centrosomes. We demonstrate that 14-3-3ε does not inhibit centriole duplication, as reported for 14-3-3γ, but inhibits centriole disengagement. Using a combination of pharmacological and genetic approaches, we demonstrate that 14-3-3ε inhibits the activity of Plk1 and separase [also known as separin (ESPL1)], leading to disengagement defects that ultimately lead to decreased proliferation and cell death. Our work demonstrates that different 14-3-3 paralogs regulate different steps in the centrosome cycle and that disrupting complex formation between 14-3-3ε and Plk1 or separase could be a novel therapeutic strategy in tumor cells.

## Teaser

**Summary:** 14-3-3ε inhibits centriole disengagement by inhibiting the activity of Plk1 and separase, resulting in either monopolar mitoses or multi-polar mitoses that result in cell death.

## INTRODUCTION

The accurate segregation of the mammalian genome relies on the formation of the mitotic spindle (Reber and Hyman, 2015). Nucleation of the microtubules to form a spindle is dependent on the centrosome, which consists of two centrioles, a mother centriole inherited from the previous cycle and a daughter centriole synthesized in the current cycle, surrounded by the pericentriolar matrix (PCM) (Bornens and Azimzadeh, 2007). Centriole duplication is initiated synchronously with DNA replication in S-phase and is limited to once per cell cycle (Hinchcliffe and Sluder, 2001). Before mitosis, the two centrosomes migrate to the two poles, nucleating a bipolar mitotic spindle (Hinchcliffe and Sluder, 2001). Centrosome amplification or a decrease in centrosome number leads to chromosomal instability and aneuploidy, which are deleterious to the cell (Basto et al., 2008; Castellanos et al., 2008; Levine et al., 2017; Pihan, 2013; Sir et al., 2013; Wong et al., 2015).

Centriole disengagement is initiated at the end of mitosis and requires the activity of the polo-like kinase 1 (Plk1) and separase [also known as separin (ESPL1)]-induced degradation of the S-M linker (a protein complex that holds the mother and daughter centriole together from S phase until the end of mitosis) or proteins required for centriole cohesion (Karki et al., 2017; Schockel et al., 2011; Tsou and Stearns, 2006; Tsou et al., 2009). During S-phase, the activity of polo-like kinase 4 (Plk4) and Cdk2 promotes procentriole biogenesis (Habedanck et al., 2005; Kleylein-sohn et al., 2007; Meraldi et al., 1999). Pro-centriole elongation and the maturation of the daughter centriole from the previous cycle into a mother centriole occurs during G2. The new mother centriole acquires distal and sub-distal appendages and the ability to nucleate PCM (Adon et al., 2010; Hinchcliffe and Sluder, 2001; Kleylein-sohn et al., 2007; Ohta et al., 2018; Whalley et al., 2015). Before mitosis, the G1-G2 tether (a protein complex that tethers the mother and daughter centriole, while new daughter centriole synthesis occurs through interphase) holding the two centrosomes together is degraded in a Plk1- and Nek2A-dependent manner (Bahe et al., 2005; Fry, 2002; Mardin et al., 2011, 2010; Yang et al., 2006a) resulting in centrosome separation and formation of a bipolar spindle (Nigg and Stearns, 2011).

The 14-3-3 protein family has seven paralogs in mammalian cells (Aitken, 2006). All 14-3-3 paralogs share a similar structure, consisting of nine α-helices that form the monomer, with each monomer in the dimer binding to a phosphorylated peptide via mode I, mode II or mode III consensus sequences (Coblitz et al., 2005; Muslin et al., 1996; Rittinger et al., 1999; Yaffe et al., 1997). Binding of 14-3-3 proteins to their ligands affects their localization, conformation, stability and function (Dalal et al., 1999; Kumagai and Dunphy, 1999; Moorhead et al., 1996; Obsil et al., 2001; Peng et al., 1997; Yaffe, 2002). This could be due to their function as molecular chaperones preventing aggregation and phase separation (Segal et al., 2023). However, it is still not clear how the loss of individual 14-3-3 paralogs leads to the phenotypes observed in mammalian cells (Dalal et al., 2004; Sehgal et al., 2014; Tilwani et al., 2021; Toyo-oka et al., 2003). Previous work from our laboratory has demonstrated that two conserved acidic amino acid residues in the phospho-peptide-binding pocket of the 14-3-3 protein family regulate complex formation and dissociation from the associated protein ligand (Bose et al., 2021; Modi et al., 2020). Altering these residues to alanine increased or decreased binding to the ligand, suggesting that these mutants might help identify specific ligands for individual 14-3-3 paralogs.

Previous work has demonstrated that 14-3-3γ and 14-3-3ε co-purify with the centrosomal fraction (Andersen et al., 2003; Pietromonaco et al., 1996), and loss of 14-3-3γ and 14-3-3ε leads to centrosome amplification in multiple cell lines (Mukhopadhyay et al., 2016). Loss of 14-3-3γ and 14-3-3ε in HaCaT cells leads to centrosome amplification, but the loss of 14-3-3γ results in clustered mitoses, whereas loss of 14-3-3ε leads to multi-polar mitoses (Tilwani et al., 2021). These results suggest that the two paralogs regulate different proteins required for centrosome licensing and duplication. Indeed, 14-3-3γ prevents centriole duplication by binding to and inhibiting NPM1 phosphorylation by Cdk2 (Bose et al., 2021). In contrast, the results in this report suggest that 14-3-3ε inhibits centriole disengagement, thereby preventing centrosome licensing. Our results identify a novel mechanism by which 14-3-3ε regulates centriole disengagement and demonstrate that different 14-3-3 paralogs affect different steps in the centrosome duplication pathway.

## RESULTS

### 14-3-3ε inhibits centriole disengagement

**Fig. 1.**
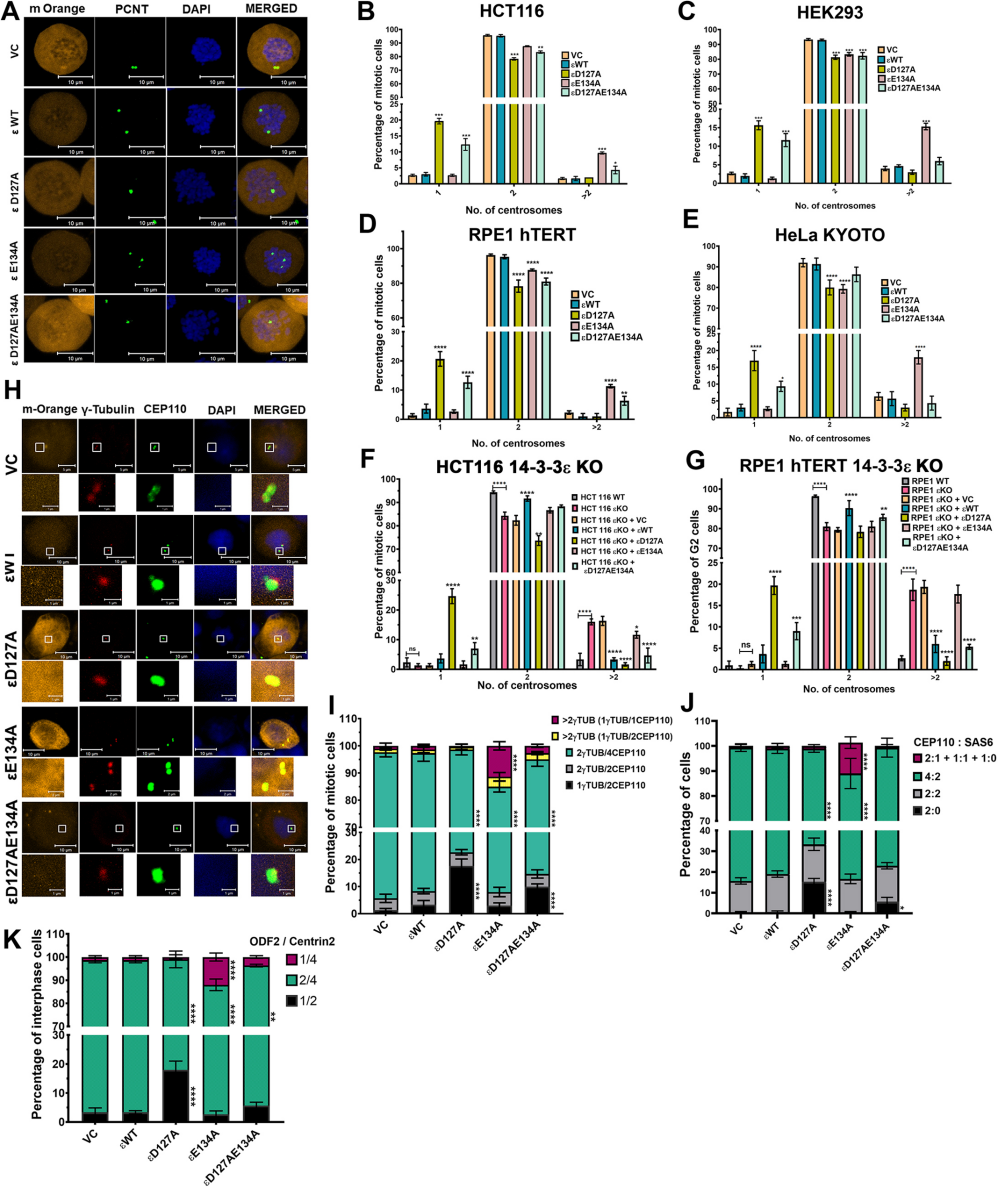
**14-3-3ε mutants alter centrosome numbers in different cell lines.** (A,B) HCT116 cells were transfected with the mOrange vector control (VC), and mOrange-tagged wild-type (WT) and mutant (D127A, E134A, D127AE134A) 14-3-3ε constructs. The cells were arrested in mitosis with nocodazole, stained with antibodies to percentrin (green) and counterstained with DAPI (blue). The number of mitotic cells showing 1, 2 or >2 centrosomes was determined in three independent experiments. Representative images are shown (A), and the mean±s.d. of three independent experiments are plotted (B). (C–G) The number of cells transfected with the indicated 14-3-3ε constructs showing 1, 2 or >2 centrosomes were determined in HEK293 (C), RPE1-hTERT (D), HELA KYOTO (E), HCT11614-3-3εKO (F) and RPE1 hTERT14-3-3εKO (G) cells. Three cell lines were arrested in prophase (C,E,F), whereas the RPE1hTERT cell lines (D,G) were arrested in G2 via use of a Cdk1 inhibitor. The mean±s.d. from three independent experiments is plotted. (H,I) HCT116 cells expressing the indicated 14-3-3ε constructs (orange) were fixed and stained with antibodies to γ-tubulin (red) and CEP110, (green) and counterstained with DAPI (blue). Representative images are shown (H). The γ-tubulin-to-CEP110 ratio was determined in three independent experiments, and the mean±s.d. are plotted (I). (J) HCT116 cells stably transfected with the VC and indicated HA14-3-3ε constructs were stained with antibodies to SAS6 and CEP110 and counterstained with DAPI. The CEP110-to-SAS6 ratio was determined in three independent experiments, and the mean±s.d. of three independent experiments were plotted. (K) HCT116 cells co-transfected with GFP–Centrin2 and the indicated 14-3-3ε constructs were stained with antibodies to ODF2 and counterstained with DAPI. The ODF2-to-Centrin2 ratio was determined in three independent experiments. *P<0.05; **P<0.01; ***P<0.001; ****P<0.0001; ns, not significant (two-way ANOVA with Tukey's multiple comparison). All comparisons are to cells transfected with the WT construct. Ratios refer to number of spots for each signal. ε, 14-3-3ε. Scale bars: 10 μm (A, H, main images other than VC); 5 μm (H, main images VC); 1 μm (H, magnifications).

We wished to determine the mechanisms by which 14-3-3ε affected centrosome duplication. As a first step, we altered the conserved aspartic and glutamic acid residues in 14-3-3ε to alanine, to give the mutations D127A and E134A, respectively, and determined the ability of these mutant proteins to affect centrosome duplication. We transfected the mOrange-tagged wild-type (WT)14-3-3ε and mutant constructs (D127A, E134A and D127AE134A) into HCT116 cells, blocked the cells in mitosis with nocodazole and stained the cells with antibodies to pericentrin to determine centrosome number. Most of the cells transfected with the vector control (VC) or WT14-3-3ε showed two centrosomes. In contrast, a significant number of cells transfected with D127A or E134A showed a single centrosome or centrosome amplification, respectively (Fig. 1A,B), a phenotype similar to that observed with similar mutants in 14-3-3γ (Bose et al., 2021). The 14-3-3γ double mutant showed a phenotype similar to that of cells expressing WT14-3-3γ (Bose et al., 2021). However, the D127AE134A mutant showed a phenotype similar to that of cells expressing D127A (Fig. 1A,B). Similar results were observed in multiple cell lines (Fig. 1C–E; Fig. S1A–C). All the mutants were expressed at equivalent levels in the different cell lines (Fig. S1D). To confirm that these results were due to changes in 14-3-3ε, we generated 14-3-3ε-knockout lines (14-3-3εKO) using Crispr/Cas9 and expressed either WT14-3-3ε or the mutant constructs in these cells. Loss of 14-3-3ε led to increased centrosome amplification in HCT116 and RPE1hTERT cells compared to the parental cells. Restoration of WT14-3-3ε reversed the phenotype, and a further decrease in centrosome amplification was observed upon expression of the D127A or D127AE134A forms. In contrast, E134A did not cause a further increase in centrosome amplification in these cells (Fig. 1F,G; Fig. S1E,F). All mutants were expressed at equivalent levels in these cells (Fig. S1G,H). The loss of another paralog, 14-3-3ζ, did not alter the centrosome number (Fig. S1I–K), suggesting that the phenotypes observed are due to the loss of 14-3-3ε.

To ensure that the defects observed are not due to the nocodazole arrest and to further characterize the centrosome phenotype, we transfected asynchronously growing HCT116 cells with the vector control, WT and mutant 14-3-3ε constructs and stained the cells with antibodies to the PCM marker γ-tubulin (Schiebel, 2000) and the centriole marker CEP110 (also known as CCP110) (Ou et al., 2002). Cells transfected with the vector control or WT constructs showed two γ-tubulin foci each containing two CEP110 foci in mitotic cells. In contrast, cells expressing D127A or D127AE134A showed an increase in the number of mitotic cells containing a single γ-tubulin focus with two CEP110 foci, and mitotic cells expressing E134A showed multiple γ-tubulin foci containing a single CEP110 focus (Fig. 1H,I). To further characterize the centrosomes, we stained HCT116 cells stably expressing HA-tagged 14-3-3ε constructs with antibodies to SAS-6 (also known as SASS6) and CEP110, which marks the site of nascent centriole formation (Banterle et al., 2021). As SAS-6 levels decrease in mitotic cells (Fong et al., 2014), we examined we were able to examine CEP110 localization in interphase cells. In cells stained with antibodies to SAS-6 and CEP110, a majority of the cells transfected with the vector control and WT constructs showed either centrosomes where centriole biogenesis had been initiated (two CEP110 foci associated with two SAS-6 foci) or cells in which centriole biogenesis had progressed (four CEP110 foci associated with two SAS-6 foci). In contrast, in the D127A- and the D127AE134A-transfected cells, a significant percentage of the cells showed two CEP110 foci with no SAS-6 staining, suggesting that centriole biogenesis had not initiated in these cells. In the E134A-transfected cells, we observed an increase in the number of cells with multiple CEP110 foci in which some centrosomes had two CEP110 foci associated with a single SAS-6 focus indicating that centriole biogenesis had progressed, whereas other CEP110 foci were either associated with one SAS-6 or no SAS-6 focus (Fig. S2A; Fig. 1J), suggesting that the mother centriole had prematurely disengaged from the daughter centriole. To further characterize the phenotype, interphase cells transfected with the mOrange-tagged 14-3-3ε constructs and GFP–Centrin2 were stained with antibodies to ODF2, which marks mother centrioles and is required to maintain centriole cohesion (Yang et al., 2018). The majority of the vector control- and WT-transfected cells showed two mature centrosomes, as illustrated by one ODF2 focus being associated with two centrin (herein assessed via centrin 2) foci, whereas the majority of the cells transfected with D127A or D127AE134A showed an increase in the number of cells with a single centrosome illustrated by the cells containing one ODF2 focus associated with two centrin foci. In contrast, a majority of the cells transfected with E134A showed the presence of one ODF2 focus associated with two centrin foci and some centrin foci that were not associated with ODF2 (Fig. S2B; Fig. 1K). These results confirm our initial observation in the nocodazole-blocked cells, which suggests that D127A inhibits centriole biogenesis and E134A promotes centrosome amplification. As the results in asynchronous cells are similar to what we observed in synchronous populations, we have used the synchrony protocols in the rest of the manuscript.

**Fig. 2.**
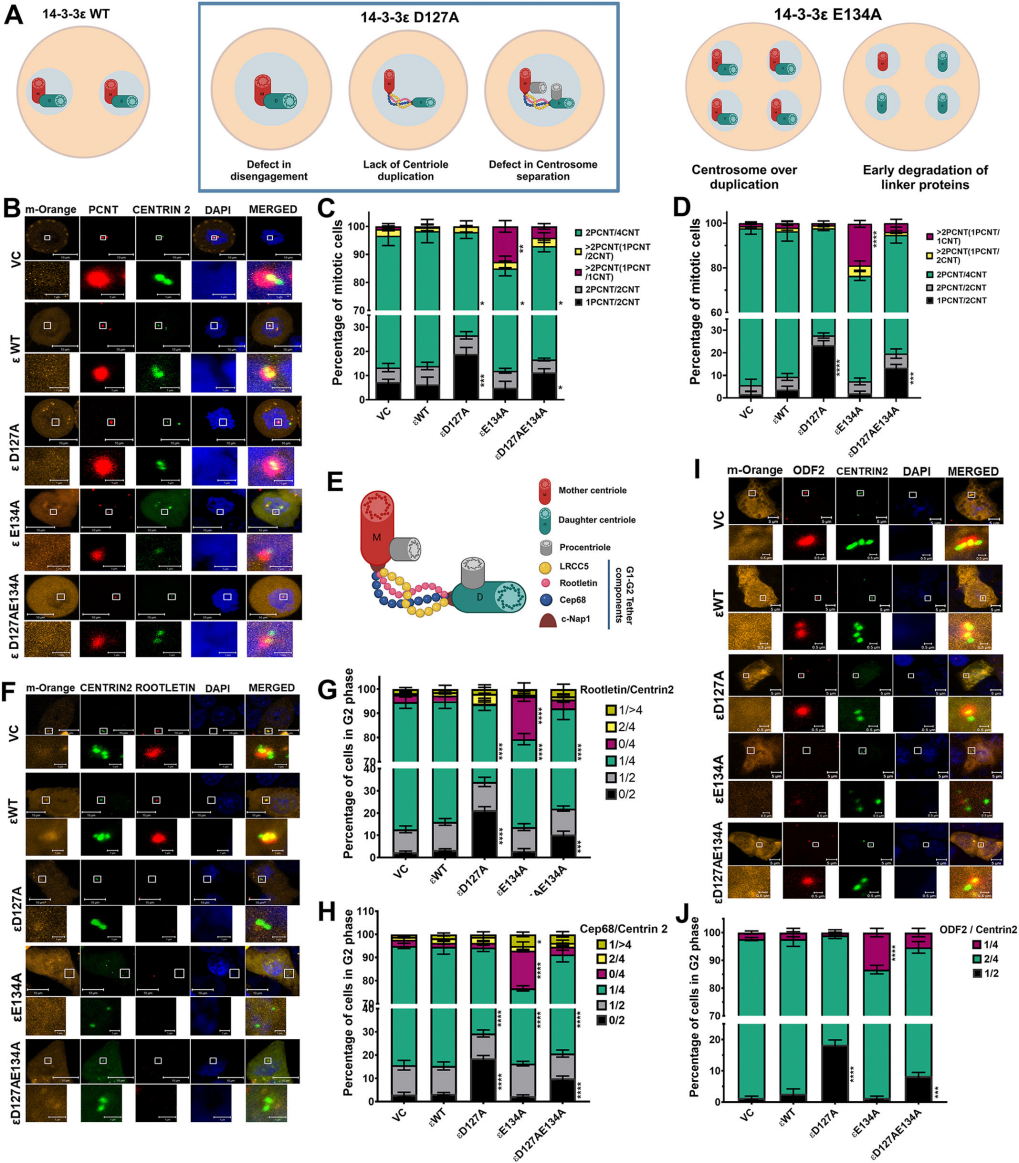
**14-3-3ε prevents centriole disengagement.** (A) Cartoon depicting the potential defect in centrosome duplication in cells expressing 14-3-3ε mutant constructs. (B–D) HCT116 and HEK293 cells were co-transfected with GFP–Centrin2 (green) and the mOrange-tagged 14-3-3ε constructs (orange), arrested in mitosis with nocodazole, and fixed and stained with antibodies to pericentrin (red) and counterstained with DAPI (blue). Representative images for HCT116 cells are shown (B). The pericentrin-to-centrin (PCNT/CNT) ratio was determined in three independent experiments for HCT116 cells (C) and HEK293 cells (D). Mean±s.d. is plotted. (E) Cartoon depicting the components and localization of the G1-G2 tether. (F,G) HCT116 cells transfected with the indicated constructs were arrested in G2 with a Cdk1 inhibitor, stained with antibodies to the G1-G2 tether protein rootletin and counterstained with DAPI. Representative images are shown (F), and the rootletin-to-centrin ratio was determined in three independent experiments, and the mean±s.d. plotted (G). (H) HCT116 cells were transfected with the indicated constructs, arrested in G2, and stained with antibodies to Cep68 and counterstained with DAPI. The Cep68-to-Centrin2 ratio was determined in three independent experiments, and the mean±s.d. plotted. (I,J) HCT116 cells were transfected with the indicated constructs, arrested in G2 and stained with antibodies to ODF2. Representative images are shown (I), and the mean±s.d. of three independent experiments plotted (J). *P<0.05; ***P<0.001; ****P<0.0001; ns, not significant (two-way ANOVA with Tukey's multiple comparison). All comparisons are to cells transfected with the WT construct. Ratios refer to number of spots for each signal. ε, 14-3-3ε. Scale bars: 10 μm (B,C, main images); 1 μm (B,C, magnifications); 5 μm (I, main images), 0.5 μm (I, magnifications).

The phenotypes observed upon expression of the different mutants could be due to defects in disengagement, defects in duplication or separation before mitosis (Fig. 2A) (Nigg and Stearns, 2011). To identify the nature of the defect, HCT116 and HEK293 cells were transfected with the 14-3-3ε constructs and GFP–Centrin2 and mitotic cells were stained with antibodies to pericentrin. All constructs were expressed at similar levels in both cell types (Fig. S2C). Most of the vector control and WT 14-3-3ε-transfected cells showed two pericentrin foci, each containing two centrin foci as expected (Fig. 2B–D; Fig. S2D). In contrast, many D127A- and D127AE134A-transfected cells showed one pericentrin foci with two centrin foci (Fig. 2B–D; Fig. S2D). In the E134A-transfected cells that showed centrosome amplification, most pericentrin foci were associated with only one centrin focus, suggesting that loss of 14-3-3ε function led to a defect in disengagement, with an absence of disengagement occurring in cells transfected with D127A and premature disengagement in cells transfected with E134A (Fig. 2B–D; Fig. S2D). The cells transfected with D127A and D127AE134A showed an increase in the intensity and area of pericentrin staining compared to cells transfected with the vector control or WT constructs (Fig. S2E,F). In contrast, cells transfected with E134A showed a decrease in the intensity and area of pericentrin staining (Fig. S2E,F). Similar phenotypes were observed when we examined cells with either a single centrosome or multiple centrosomes in different mitotic phases (Fig. S2G,H). This phenotype is distinct from the results we observed with 14-3-3γ, where a defect in centriole duplication was observed (Bose et al., 2021).

Centriole disengagement requires degradation of the S-M linker or proteins that maintain centriole cohesion, at the end of mitosis and the beginning of G1, followed by the formation of the G1-G2 tether and subsequent centriole duplication (Fig. 2E; Nigg and Stearns, 2011). To determine whether the G1-G2 tether had formed in these cells, we transfected the 14-3-3ε constructs along with GFP–Centrin2 into these cells, arrested them in G2 by treatment with a Cdk1 inhibitor and stained with antibodies to different G1-G2 tether proteins. The 14-3-3ε constructs were expressed at equivalent levels in the transfected cells (Fig. S2I). Cells expressing the vector control or WT 14-3-3ε showed that the GFP–Centrin2 foci colocalized with staining for the G1-G2 tether markers rootletin (CROCC) and Cep68 (Bahe et al., 2005; Graser et al., 2007) (Fig. 2F–H; Fig. S2J). In contrast, in cells expressing D127A and D127AD134A, a pair of centrin foci did not colocalize with the G1-G2 tether markers, whereas in cells expressing E134A, the single centrin foci were not localized with the G1-G2 tether markers (Fig. 2F–H; Fig. S2J). Staining with antibodies to the mother centriole-specific marker ODF2 in G2-arrested cells (Fig. 2I–J) gave similar results to those observed in the asynchronously growing cells (Fig. S2B; Fig. 1K), suggesting that the events observed occur in G2 phase. All the transfected cells showed equivalent levels of the WT and mutant 14-3-3ε proteins (Fig. S2I). To determine the cell cycle phase in which disengagement occurs, we synchronized the stable cell lines in late G1 with mimosine (Dalal et al., 1999). The cells were released from mimosine, harvested at different time points and the cell cycle distribution was determined (Fig. S3A) followed by immunofluorescence analysis with antibodies to Cep110 and rootletin (Fig. S3B–E). In G1 cells, a significant percentage of cells expressing D127A showed two Cep110 foci that were not associated with rootletin staining, in contrast to cells expressing the other constructs, indicating that disengagement had not occurred (Fig. S3B,C). In S and G2 cells expressing E134A, we observed multiple Cep110 foci that were not associated with rootletin staining (Fig. S3D,E). The D127A phenotype persisted during S–G2 (Fig. S3D,E). Cells in G2 and M were stained with antibodies to Cep110 and pericentrin. Cells expressing D127A showed a single pericentrin focus with two Cep110 foci whereas cells expressing E134A showed cells with multiple pericentrin foci containing a single Cep110 foci (Fig. S3F,G). Taken together, these results suggest that 14-3-3ε inhibits centrosome disengagement.

### The single and multiple centrosomes observed in the 14-3-3ε mutants anchor microtubules but show mitotic defects

**Fig. 3.**
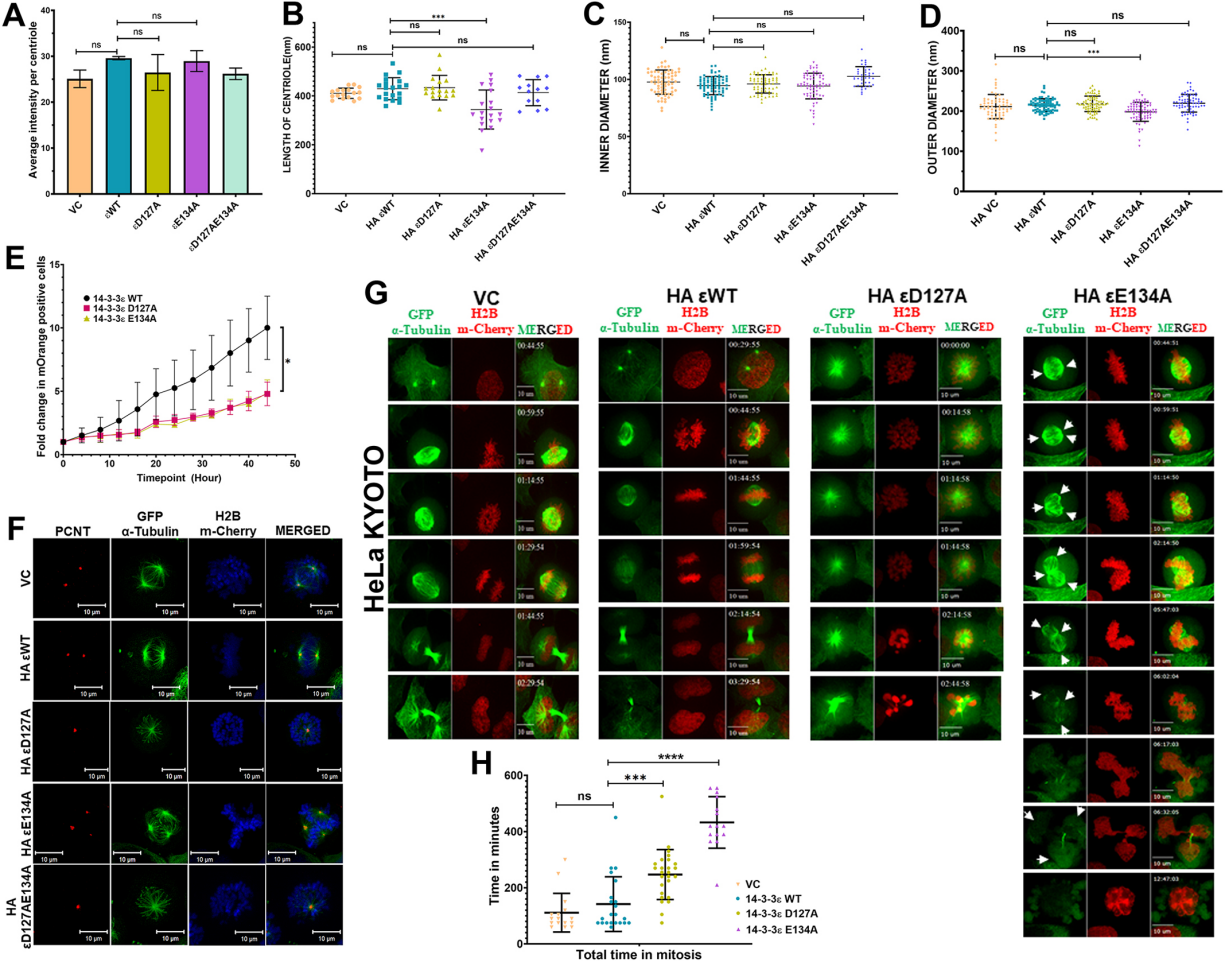
**Expression of the 14-3-3ε mutants leads to a mitotic delay and cell death.** (A) HCT116 cells transfected with the indicated constructs were arrested in mitosis and stained with antibodies to glutamylated tubulin (GT335), and the signal intensity was measured. The mean±s.d. from three independent experiments are plotted. (B–D) HCT116 cells stably expressing the vector control (VC) and HA-tagged 14-3-3ε constructs were synchronized in G2-M phase and processed for transmission electron microscopy. The length of the centriole (*n*>15) (B), the inner diameter of the centriole (*n*>39) (C), and the outer diameter of the centriole (*n*>69) (D) were measured in three independent experiments and the mean±s.d. plotted. (E) The proliferation of HCT116 cells expressing the vector control and mOrange-tagged 14-3-3ε constructs was measured in an Incucyte live cell analysis system. Images of the same field were acquired every hour, and the mean±s.d. of the normalized counts (mOrange positive/total number of cells) per field from three independent experiments were plotted against time (hours). (F) HeLa KYOTO cells stably transfected with the vector control and HA-tagged 14-3-3ε constructs were arrested in mitosis and stained with antibodies against pericentrin (red). The cells express GFP α-tubulin (green) and H2B–mCherry (blue). (G,H) HeLa KYOTO cells stably expressing the vector control and HA-tagged 14-3-3ε constructs were imaged over 24 h in an Olympus 3i spinning disc microscope. Representative images of the same cell acquired over the period and stained for GFP–α-tubulin (green) and H2B–mCherry (red) are shown with the time stamp in the upper left corner. The arrowheads indicate microtubule-organizing centers, showing the premature disengagement of centrioles (G). The mean±s.d. of the time spent in mitosis of ≥15 cells across three independent experiments is plotted (H). Note that in contrast to the VC or WT-expressing cells, the D127A cells never complete mitosis, and the E134A cells undergo multipolar mitosis. *P<0.05; ***P<0.001; ****P<0.0001; ns, not significant [Student's unpaired *t*-test with Welch's correction (A); one-way ANOVA with Tukey's multiple comparison (B–E,H)]. All comparisons are to cells transfected with the WT construct. ε, 14-3-3ε. Scale bars: 10 μm.

Injecting cells with a monoclonal antibody to glutamylated tubulin can result in centriole fragmentation (Bobinnec et al., 1998). To determine whether the centrioles observed in the transfected cells had alterations in the levels of glutamylated tubulin, we performed staining with an antibody to glutamylated tubulin, GT335 (Bobinnec et al., 1998). The 14-3-3ε proteins are present at equivalent levels (Fig. S4A), and the levels of glutamylated tubulin at the centriole in cells transfected with the vector control or the WT or mutant 14-3-3ε constructs did not show significant differences (Fig. S4B; Fig. 3A) suggesting that any defects observed were not due to changes in the levels of glutamylated tubulin at the centrosome. We then generated stable lines expressing the WT and mutant 14-3-3ε constructs in HCT116 cells (Fig. S3A). We measured centriole length and the inner and outer diameter of the centriole using electron microscopy in cells arrested in G2 phase with the Cdk1 inhibitor, as our previous experiments demonstrated that the centrosome phenotype was not affected by treatment with the Cdk1 inhibitor (Fig. S4C). Expression of WT 14-3-3ε or D127A and D127AE134A did not result in a significant change in these phenotypes as compared to the vector control (Fig. 3B–D). In contrast, the centrioles were significantly shorter in cells expressing E134A and also had a smaller outer diameter than in cells expressing WT 14-3-3ε (Fig. 3B,D). No change in the inner diameter of the centriole was observed in these cells (Fig. 3C). Whereas only ∼20% of the E134A-transfected cells showed the multiple centrosome phenotype constructs (Fig. 1A,B), all the E134A-transfected cells showed shorter centrioles as compared to the other constructs (Fig.3B–D), suggesting a defect in the centriole maturation in these cells (Sullenberger et al., 2020). To determine whether these altered centrosome phenotypes resulted in a change in proliferation, the mOrange-tagged WT and mutant constructs were transfected into cells, and the number of mOrange-positive cells was monitored over time. The number of mOrange-positive cells at each time point was normalized to the initial number of mOrange-positive cells present at the start of the experiment for each construct. Cells expressing WT 14-3-3ε showed a higher growth rate than cells expressing the D127A and E134A (Fig. 3E).

To determine whether these centrosomes could anchor microtubules, we transfected the mOrange-tagged WT and mutant 14-3-3ε constructs into HCT116 cells and stained them with antibodies to pericentrin and α-tubulin. Cells transfected with the vector control or WT 14-3-3ε showed typical bipolar spindles in mitotic cells. In contrast, cells transfected with D127A and D127AE134A showed the presence of monopolar spindles, whereas cells transfected with E134A showed multipolar spindles (Fig. S4D). To further examine the mitotic phenotypes resulting from the expression of these mutants, we stably transfected the WT and mutant 14-3-3ε constructs into HeLa Kyoto cells that expressed EGFP–tubulin and H2B–mCherry (Fig. S4A; Bastos and Barr, 2010). The transfected cells were stained with antibodies to pericentrin. Cells transfected with the vector control or WT 14-3-3ε showed typical bipolar spindles in mitotic cells. In contrast, cells transfected with D127A and D127AE134A showed the presence of monopolar spindles, whereas cells transfected with E134A showed multipolar spindles (Fig. 3F). We then used these stably transfected cells to determine mitotic outcomes in live-cell imaging experiments. Cells expressing WT 14-3-3ε completed mitosis in ∼80 min (Fig. 3G,H; Movies 1, 2). In contrast, cells expressing D127A that showed mono-polar spindles never exited prophase and ultimately died in prometaphase, whereas cells expressing E134A that showed multi-polar mitoses completed mitosis in ∼280 min followed by cell death in G1 or died due to a prolonged metaphase arrest (Fig. 3G,H; Movies 3, 4). These results are consistent with our observations that cells expressing these mutants show a decrease in proliferation (Fig. 3E), which is probably because a significant percentage of cells with a single centrosome die in prophase whereas the cells undergoing multi-polar mitosis also die as previously described (Ganem et al., 2009).

### 14-3-3ε inhibits centriole disengagement by inhibiting the activity of Plk1 and separase

**Fig. 4.**
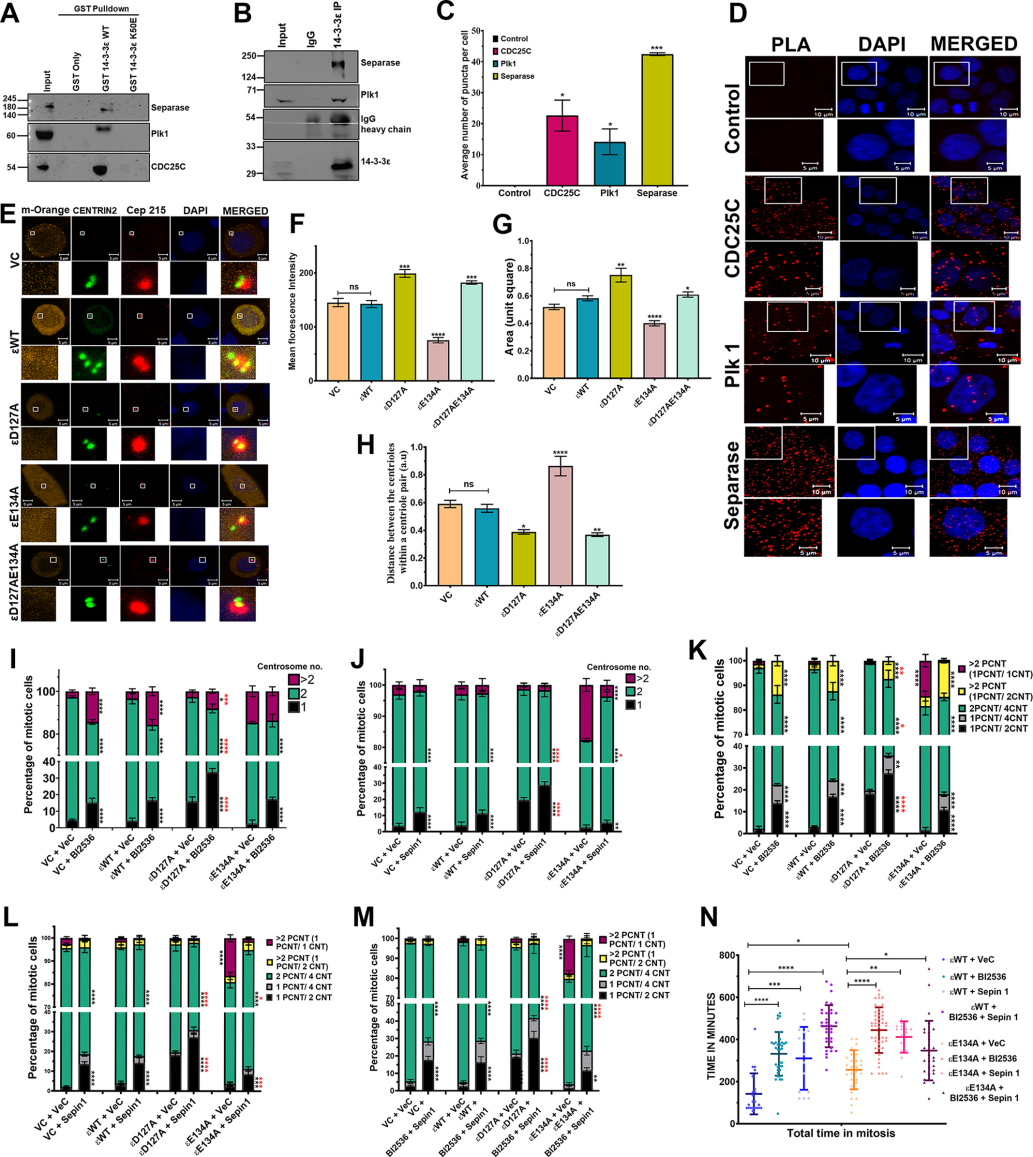
**14-3-3ε forms a complex with Plk1 and separase.** (A) Protein extracts prepared from HCT116 cells were incubated with the indicated GST fusion proteins, and the reactions resolved on SDS-PAGE gels, followed by western blotting with the indicated antibodies. Input, 10%. (B) Protein extracts prepared from HCT116 cells were incubated with either a non-specific IgG or antibodies to 14-3-3ε, and the reactions resolved on SDS-PAGE gels followed by western blotting with the indicated antibodies. Input, 10%. Blots shown representative of three repeats. (C,D) PLAs were performed in HCT116 cells, using antibodies against 14-3-3ε and either Plk1, separase and cdc25C. Representative images are shown (D), and the mean±s.d. of the number of puncta per cell from three independent experiments are plotted (C). (E–H) HCT116 cells overexpressing GFP–Centrin2 and mOrange or mOrange-tagged 14-3-3ε constructs were arrested in mitosis and stained with antibodies to Cep215 and counter stained with DAPI. Representative images are shown (E), and the mean±s.e.m. from three independent experiments for the intensity of Cep215 staining (F), centrosome size (G) and distance between the centriole pair (H) pair is plotted. (I,J) Cell lines were transfected with mOrange vector control or mOrange-tagged 14-3-3ε constructs and were treated with BI2536 and/or Sepin1 or the vehicle control (DMSO). The mean±s.d. of the number of centrosomes in mitotic cells from three independent experiments are plotted for cells treated with BI2536 (I) or Sepin-1 (J). (K–M) HCT 116 cells were transfected with the mOrange vector control (VC) or mOrange-tagged 14-3-3ε constructs and GFP–Centrin2, and were treated with BI2536, Sepin-1, both BI2356 and Sepin-1, or the vehicle control (DMSO). The mean±s.d. of the pericentrin to centrin2 ratio from three independent experiments is plotted for BI2536 (K), Sepin-1 (L) and BI2536 and Sepin1 (M). (N) HeLa KYOTO cells stably expressing HA-tagged 14-3-3ε WT or 14-3-3ε E134A were treated with vehicle control (DMSO), BI2536, Sepin-1 or both BI2536 and Sepin-1, and were imaged for 20 h at intervals of 20 min. The mean±s.d. from ≥15 cells across three independent experiments are plotted. *P<0.05, **P<0.01, ***P<0.001; ****P<0.0001; ns, not significant [unpaired Student's *t*-test with Welch's correction (C, F–H); one-way ANOVA Tukey's multiple comparison (I–N; black asterisks refer to comparison within the group with respective vehicle control, red asterisks refer to comparison across the group with 14-3-3ε WT treated with drug)]. Ratios refer to number of spots for each signal. ε, 14-3-3ε. Scale bars: 10 μm (D, main images); 5 μm (D, magnifications, E).

The activity of Plk1 and separase induce degradation proteins required for centrosome cohesion (Karki et al., 2017; Schockel et al., 2011; Tsou and Stearns, 2006; Tsou et al., 2009) and are required for centrosome licensing and duplication. As the defect observed in cells transfected with the 14-3-3ε mutants seemed to be a lack of cohesion or premature disengagement, we asked whether 14-3-3ε formed a complex with Plk1 or separase. GST pulldown assays demonstrated that GST–14-3-3ε formed a complex with both Plk1 and separase in contrast to GST alone or the ligand binding defective mutant GST–14-3-3εK50E (Brunet et al., 2002) (Fig. 4A; Fig. S5A). Similarly, co-immunoprecipitation experiments in HCT116 cells demonstrated that 14-3-3ε formed a complex with both Plk1 and separase (Fig. 4B; Fig. S5B,C). This was true whether the antibody used for immunoprecipitation was raised against 14-3-3ε, Plk1 or separase. Proximity ligation assays (PLAs) also demonstrated that 14-3-3ε formed a complex with both Plk1 and separase in cells, where Cdc25C serving as a control (Fig. 4C,D). These results suggest that 14-3-3ε inhibits the function of Plk1 and separase, thereby preventing premature degradation of the S-M linker or proteins required for centriole cohesion. If this hypothesis is correct, one prediction would be that the levels of Cep215 (also known as CDK5RAP2), whose degradation occurs before disengagement (Graser et al., 2007; Pagan et al., 2015), would be altered in cells expressing the different 14-3-3ε mutants. When we stained cells transfected with WT or mutant 14-3-3ε constructs and stained them with antibodies to Cep215, we observed that the intensity of Cep215 staining at the centrosome was highest in cells expressing D127A as compared to WT 14-3-3ε (Fig. 4E,F). In contrast, cells expressing E134A showed decreased staining at the centrosome for Cep215 (Fig. 4E,F). Consistent with these observations, centrosome size as measured by Cep215 staining was elevated in cells expressing the D127A mutant (Fig. 4G). In contrast, the distance between the two centrioles was elevated in cells expressing E134A (Fig. 4H). These are consistent with our data on pericentrin staining (Fig. S2E–H) and also with previous observations showing that Cep215 and pericentrin degradation are required for centriole disengagement and licensing (Pagan et al., 2015).

As Plk1 and separase are required for the degradation of the S-M linker and proteins required for centriole cohesion, we asked whether the small-molecule inhibitors of Plk1 and separase, BI2536 (Lenart et al., 2007; Steegmaier et al., 2007) and Sepin-1 (Zhang et al., 2014), respectively, could alter the phenotypes observed upon expression of the 14-3-3ε constructs. Treatment of the cells with the inhibitors did not alter the levels of the transfected constructs (Fig. S5D). The cell cycle profile of the treated cells is shown in Fig. S5E. Treatment of cells with the Plk1 inhibitor BI2536 led to a significant increase in the percentage of cells with a single centrosome compared to the vehicle control (Fig. 4I). We also observed an increase in the percentage of cells with more than two centrosomes, which might be due to a failure of cytokinesis followed by centrosome duplication in the subsequent cycle, as previously reported (Kasahara et al., 2013) (Fig. 4I). Treatment with Sepin-1 resulted in an increase in the percentage of cells with a single centrosome in cells transfected with D127A and a decrease in the percentage of cells showing centrosome amplification in cells transfected with E134A (Fig. 4J). When we examined the pericentrin-to-centrin ratio in cells treated with BI2356, we observed that treatment with BI2356 led to a significant decrease in disengagement as the number of cells with only one pericentrin focus and two centrin foci, as well as cells with one pericentrin focus and four pericentrin foci increased significantly, whereas the number of cells with one pericentrin focus containing a single centrin spot decreased significantly with a concomitant increase in cells with normal centrosome number in the E134A-transfected cells (Fig. 4K; Fig. S5F). Similar results were observed when we treated cells with Sepin-1 (Fig. 4L; Fig. S5G). A small additive effect was observed when cells were treated with both inhibitors (Fig. 4M; Fig. S5H), which is consistent with the observation that Plk1 activity is required to promote the cleavage of substrates by separase (Karki et al., 2017; Schockel et al., 2011; Tsou and Stearns, 2006; Tsou et al., 2009). Similar results were observed when we treated the 14-3-3εKO cells with either inhibitor or a combination of the two inhibitors (Fig. S5I,J). One possible prediction from these experiments is that treatment with the inhibitors would extend the time spent in mitosis. We treated the HeLa lines stably expressing the WT and E134A with either BI2536 or Sepin-1 or both inhibitors, followed by live-cell imaging (Fig. S5K–M; Movies 5–12). The amount of time spent in mitosis was significantly increased for cells that expressed both WT 14-3-3ε and the E134A (Fig. 4N). These results suggest that 14-3-3ε might regulate the function of both Plk1 and separase.

**Fig. 5.**
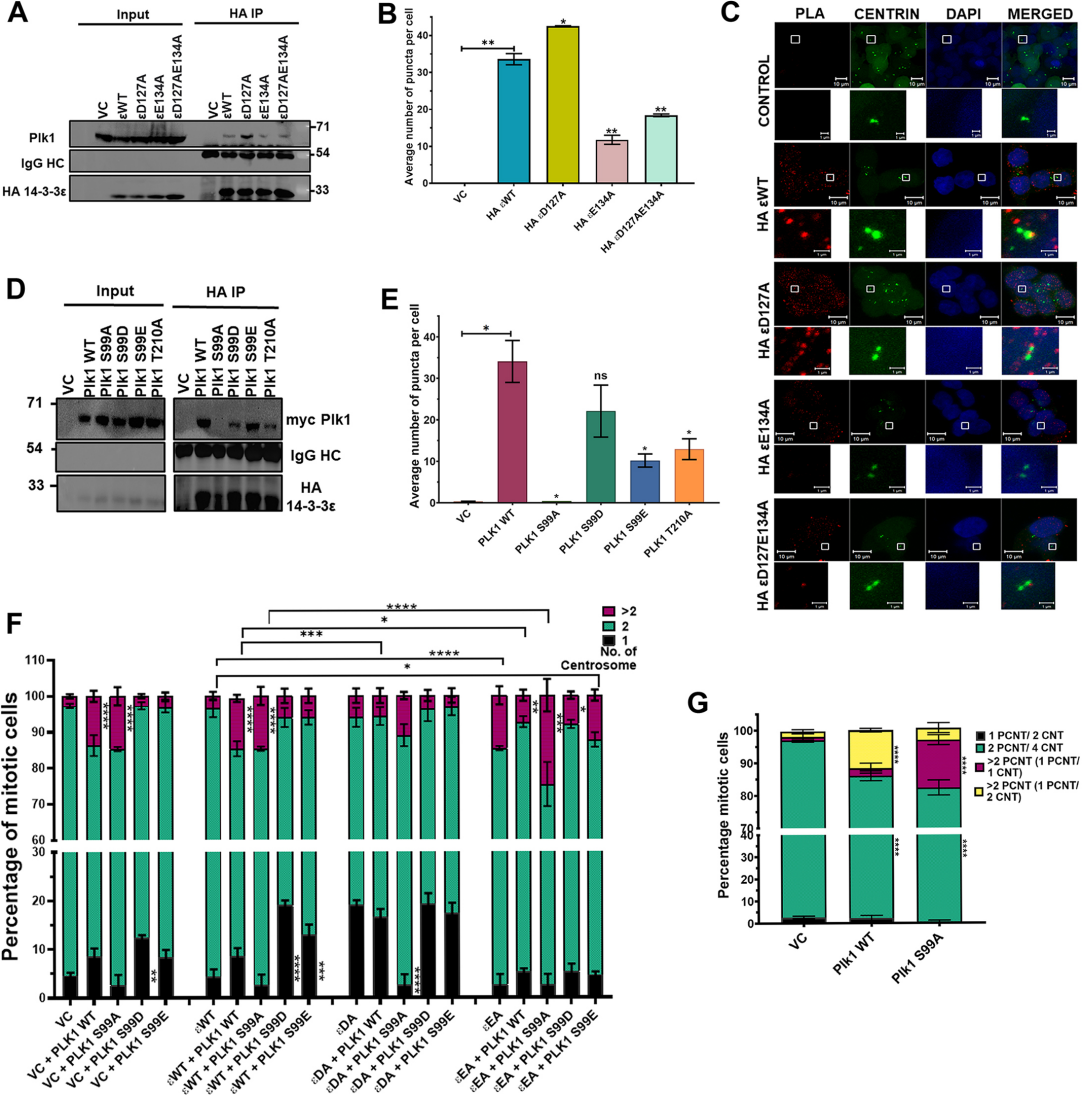
**14-3-3ε inhibits Plk1, preventing centriole disengagement.** (A) Protein extracts prepared from HCT116 cells transfected with vector control or HA-tagged 14-3-3ε constructs were incubated with antibodies against HA, and the reactions resolved on SDS-PAGE gels followed by western blotting with the indicated antibodies. Input, 10%. Blot shown representative of three repeats. (B,C) HCT116 cells stably expressing GFP–Centrin2 were transfected with the indicated constructs and PLA assays performed using antibodies against HA and Plk1. Representative images are shown in C. PLA, red; DAPI, blue. The mean±s.d. number of puncta per cell for three independent experiments is plotted (B). (D,E) HCT116 cells expressing HA-tagged 14-3-3ε WT were co-transfected with GFP–Centrin2 and Myc-tagged WT Plk1 mutant constructs (S99A, S99D, S99E and T210A). Protein extracts were prepared from these cells, and immunoprecipitations were performed with antibodies to HA, followed by western blotting with the indicated antibodies. Blot shown representative of three repeats (D). PLAs using antibodies against the HA and Myc epitopes were performed, and the mean and standard deviation of the number of puncta per cell for three independent experiments were plotted (E). (F) HCT116 cells stably expressing vector control and HA-tagged 14-3-3ε constructs were transfected with Myc-tagged Plk1 WT and mutant constructs (S99A,S99D and S99E) followed by staining with antibodies against the Myc epitope tag and pericentrin. The centrosome number in mitotic cells was determined in three independent experiments, and the mean±s.d.were plotted. (G) HCT116 cells stably expressing the vector control, Myc-tagged Plk1 WT and the Plk1 S99A mutant were stained with antibodies to the Myc epitope tag, Centrin2 and pericentrin and the PCNT/CNT ratio was determined in three independent experiments, and the mean±s.d. plotted. *P<0.05; **P<0.01; ***P<0.001; ****P<0.0001 [unpaired Student's *t*-test with Welch's correction (B,E); two-way ANOVA with Tukey's multiple comparison (G)]. Ratios refer to number of spots for each signal. ε, 14-3-3ε. Scale bars: 10 μm (main images); 1 μm (magnifications).

Previous experiments have demonstrated that Plk1 can bind to 14-3-3ζ when phosphorylated at serine 330 or 597 and 14-3-3γ when phosphorylated at serine 99 (Du et al., 2012; Kasahara et al., 2013). As a first step to determine whether 14-3-3ε regulated Plk1 function, we determined the interaction of the WT and 14-3-3ε mutants with Plk1. Co-immunoprecipitation and GST pulldown assays demonstrated that D127A showed a greater interaction with Plk1 than WT14-3-3ε. In contrast, E134A bound to Plk1 at a lower efficiency than WT (Fig. 5A; Fig. S6A). Similar results were observed with Cdc25C (Fig. S6A). The changes in the association of the 14-3-3ε mutants with Plk1 were not due to a change in their overall structure as determined by their circular dichroism (CD) spectra (Fig. S6B). Similar results were observed in PLA assays, demonstrating that the interaction occurred at the centrosome (Fig. 5B,C). We then altered a potential 14-3-3-binding site in Plk1 at S99 to alanine (S99A), aspartic acid (S99D) and glutamic acid (S99E) and determined the interaction of these mutants with 14-3-3ε. All the mutants were expressed at equivalent levels (Fig. S6C). Co-immunoprecipitation assays demonstrated that S99A failed to bind to 14-3-3ε, unlike S99D and S99E, which serve as phospho-mimetic mutants (Fig. 5D). As controls, we altered another potential 14-3-3-binding site, threonine 210 to Alanine (T210A) and demonstrated that it formed a complex with 14-3-3ε (Fig. 5D). Similar results were obtained in PLA assays (Fig. 5E; Fig. S6D). We then evaluated the ability of these mutants to affect centrosome number. The expression of Plk1S99A resulted in a significant decrease in the percentage of cells with a single centrosome in cells expressing D127A. In contrast, the phospho-mimetic mutants (S99D and S99E) and WTPlk1 did not significantly alter the percentage of cells with a single centrosome (Fig. 5F; Fig. S6E). In cells expressing E134A, Plk1S99A stimulated a further increase in the percentage of cells showing supernumerary centrosomes as compared to WTPlk1 or S99D and S99E (Fig. 5F; Fig. S6E). To determine the role of these Plk1 mutants in regulating centriole disengagement, the WT or mutant Plk1 constructs were transfected into HCT116 cells, and the pericentrin-to-centrin ratio was determined in cells arrested in mitosis. The expression of WTPlk1 led to a significant increase in centrosome amplification compared to the vector control. In contrast, expression of S99A led to premature disengagement, as illustrated by cells containing an increased number of pericentrin foci with just one centrin dot (Fig. 5G; Fig. S6F). To further confirm the observation that 14-3-3ε inhibits Plk1 function, we determined the levels of Plk1 phosphorylated on T210, which stimulates Plk1 kinase activity (Bruinsma et al., 2015). Cells expressing the vector control or WT14-3-3ε showed equivalent levels of phospho-Plk1 staining in mitotic cells. In contrast, cells expressing D127A or E134A showed minimal or enhanced staining for phospho-Plk1 respectively (Fig. S6G,H). These results suggest that 14-3-3ε inhibits centrosome disengagement by suppressing Plk1 activity.

**Fig. 6.**
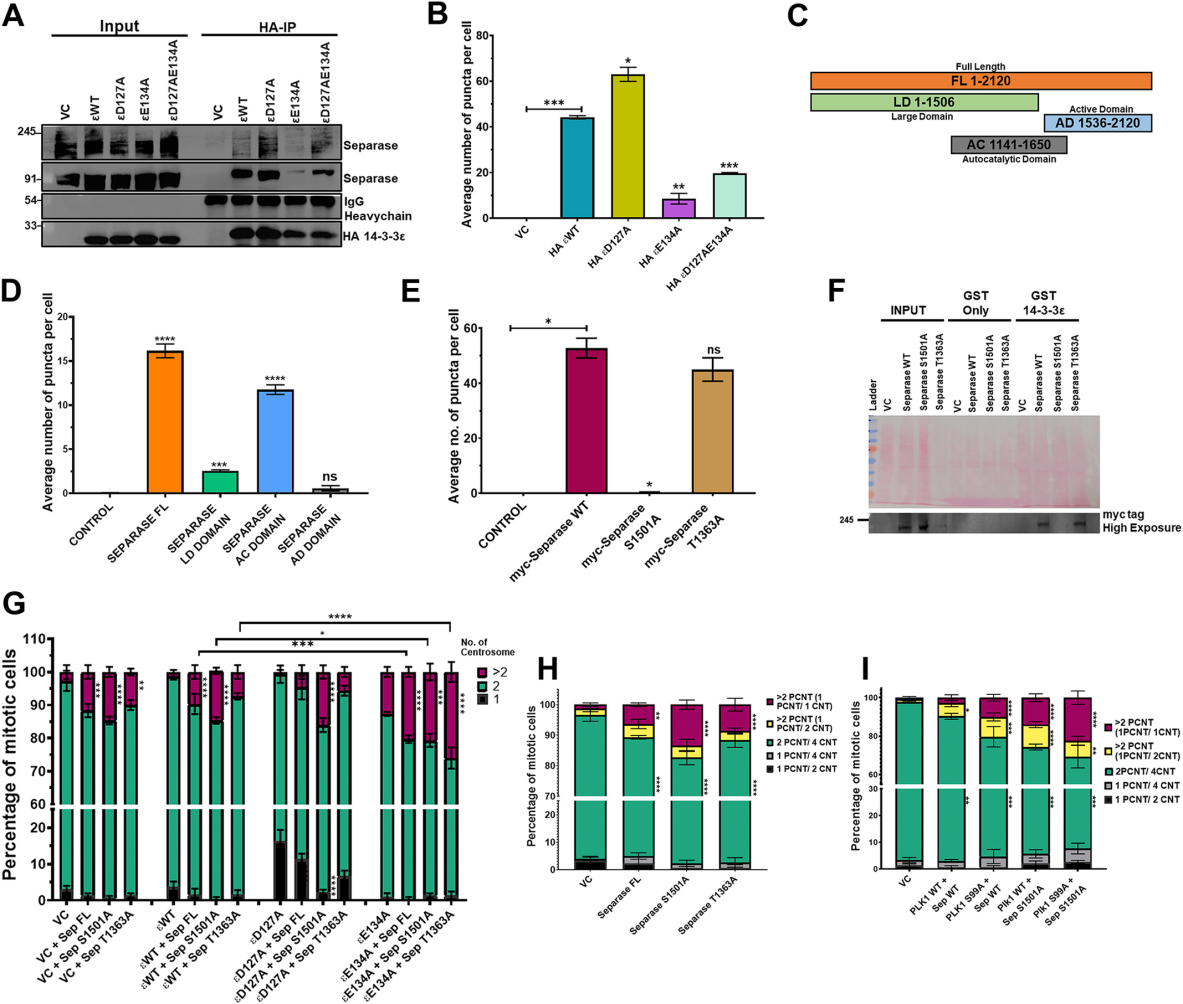
**14-3-3ε inhibits separase function, inhibiting centriole disengagement.** (A) Protein extracts prepared from HCT116 cells transfected with the indicated constructs were incubated with antibodies to HA, and the reactions resolved on SDS-PAGE gels followed by western blotting with the indicated antibodies. Blot shown representative of three repeats. (B) HCT116 cells stably expressing GFP–Centrin2 were transfected with the indicated constructs and PLA assays performed using antibodies against HA and separase. The number of PLA puncta per cell was determined in three independent experiments, and the mean±s.d. plotted. (C) Diagram of the separase deletion mutants. (D) Protein extracts were prepared from HCT116 cells transfected with Myc-tagged separase (FL) and Myc-tagged deletion mutants [large domain (LD), auto-catalytic domain (AC), and active domain (AD)]. PLA assays were performed with antibodies to Myc and 14-3-3ε, and the number of PLA puncta per cell was determined in three independent experiments, and the mean±s.d. plotted. (E) HCT116 cells stably expressing GFP–Centrin2 were transfected with Myc-tagged separase WT and mutant constructs (S1501A and T1363A), and PLA assays were performed with antibodies to the Myc epitope tag and 14-3-3ε. The number of PLA puncta per cell was determined in three independent experiments, and the mean±s.d. plotted. (F) Protein extracts prepared from HCT116 cells were transfected with Myc-tagged separase WT mutant constructs (S1501A and T1363A) were incubated with the indicated GST fusion proteins followed by western blotting with the indicated antibodies. Input. 10%. Blot shown representative of three repeats (G) HCT116 cells stably expressing vector control and HA-tagged 14-3-3ε constructs were transfected with Myc-tagged separase WT, and Myc-tagged separase (S1501A andT1363A) constructs, the cells arrested in mitosis followed by staining with antibodies to Myc and pericentrin. The mean±s.d. of the centrosome number for three independent experiments is plotted. (H) HCT116 cells stably expressing GFP–Centrin2 were transfected with Myc-tagged separase WT and mutant constructs (S1501A and T1363A) and stained with antibodies against the Myc epitope tag, pericentrin and the PCNT/CNT ratio was determined was determined in three independent experiments. The mean±s.d. is plotted. (I) HCT116 cells stably expressing Myc-tagged Plk1 and Myc-tagged Plk1 S99A were transfected with Myc-tagged separase, and Myc-tagged separase S1501A mutant ans stained with antibodies against the Myc epitope tag, pericentrin and Centrin2, and the PCNT/CNT ratio determined in three independent experiments. Mean±s.d. plotted. *P<0.05; **P<0.01; ***P<0.001; ****P<0.0001; ns, not significant [unpaired *t*-test with Welch's correction (B,D,E) and two-way ANOVA with Tukey's multiple comparison (G–I)]. Ratios refer to number of spots for each signal. ε, 14-3-3ε; Sep, separase.

As 14-3-3ε forms a complex with separase and treatment with Sepin-1 phenocopies the effect of D127A (Fig. 4), we determined the ability of the WT and mutant 14-3-3ε constructs to form a complex with separase. Co-immunoprecipitation assays demonstrated that D127A formed a complex with separase with greater efficiency than WT14-3-3ε (Fig. 6A). In contrast, E134A failed to form a complex with separase (Fig. 6A). Similar results were observed in PLA assays (Fig. 6B; Fig. S7A,B). To identify the 14-3-3ε-binding site in separase, we generated deletion mutants of separase (Fig. 6C) and tested their ability to bind 14-3-3ε in PLA assays. WT separase and a mutant containing just the autocatalytic domain (amino acids 1141–1650) were in close proximity to 14-3-3ε, while the N-terminal and C-terminal domains of separase [the LD (large domain) and AD (active domain), respectively] did not form a stable complex with 14-3-3ε (Fig. 6D; Fig. S7C,D). Using the 14-3-3Pred software (https://www.compbio.dundee.ac.uk/1433pred), we identified two potential 14-3-3-binding sites in the AC domain, threonine 1363 (T1363) and serine 1501 (S1501), altered them to alanine in full-length separase (T1363A and S1501A) and tested their ability to bind to 14-3-3ε in PLA and GST pulldown assays. The PLAs demonstrated that T1363A formed a complex with 14-3-3ε at levels similar to WTseparase. However, S1501A failed to bind to 14-3-3ε in these assays (Fig. 6E; Fig. S7E,F). Similar results were obtained in the GST pulldown assays (Fig. 6F). Next, we determined the effect of expressing the separase mutants on the centrosome number. Expression of WTseparase or T1363A led to a significant increase in the number of cells with more than two centrosomes when co-transfected with WT14-3-3ε or D127A and E134A (Fig. 6G; Fig. S7G). Expression of S1501A led to a significant increase in the number of cells with more than two centrosomes and a huge decrease in the number of cells with a single centrosome in cells expressing D127A (Fig. 6G; Fig. S7G). When we examined the organization of centrosomes in the cells transfected with the different separase mutants, we observed that the cells with more than two centrosomes had just one centrin focus in one pericentrin focus and that this number was greatly elevated in cells transfected with the separaseS1501A (Fig. 6H; Fig. S7H). Finally, co-expression of the WT and/or the 14-3-3ε binding defective mutants of separase and Plk1 led to a significant increase in the number of cells with more than two centrosomes (Fig. 6I; Fig. S7I). Expression of either the Plk1 or separase mutant led to an increase in the number of cells with more than two centrosomes that had just one centrin focus in one pericentrin focus. This number was greatly elevated in cells transfected with both mutant constructs, suggesting a cumulative effect of the expression of both mutant proteins (Fig. 6I; Fig. S7I). These results demonstrate that 14-3-3ε regulates the activity of both Plk1 and separase to inhibit premature centriole disengagement in the centrosome duplication cycle.

## DISCUSSION

The results in this report indicate that 14-3-3ε prevents premature centriole disengagement by inhibiting the function of both Plk1 and separase. Increased complex formation between 14-3-3ε and Plk1 or separase leads to defects in disengagement post mitosis, thereby preventing centrosome licensing centriole duplication and monopolar spindle formation, resulting in cell death. A decrease in complex formation between 14-3-3ε and Plk1 or separase leads to premature cleavage of the S-M linker or proteins required for centriole cohesion in interphase or early mitosis, resulting in single centrioles and multi-polar mitoses, resulting in cell death.

Multiple reports have suggested that the loss of specific 14-3-3 paralogs leads to different phenotypes in cell lines (Dalal et al., 2004; Rietscher et al., 2018; Telles et al., 2009; Tilwani et al., 2021; Valente et al., 2012) and in animal models (Cheah et al., 2012; Sehgal et al., 2014; Steinacker et al., 2005; Tilwani et al., 2024; Toyo-oka et al., 2003, 2014). Furthermore, structural features shared by 14-3-3ε and 14-3-3γ regulate complex formation with the mitotic phosphatase Cdc25C (Telles et al., 2009). Recent proteomic studies that wished to identify paralog-specific interactors suggested that other than 14-3-3σ, the other 14-3-3 paralogs bound to a similar set of ligands but with different affinities, suggesting that the differences between different 14-3-3 paralogs was in their affinity for the ligand rather than specific association with the ligand (Segal et al., 2023). However, these studies were confounded by the fact that unlike 14-3-3σ, which exclusively forms homodimers (Wilker et al., 2005), all the other paralogs form both homodimers and heterodimers (Yaffe et al., 1997; Yang et al., 2006b), and therefore, the low-affinity interactions might be because the target ligand might be binding to another paralog in the dimer. As 14-3-3–ligand complexes are potential drug targets, small molecules that promote disruption or formation of these complexes could serve as novel therapeutics. However, before small molecules that disrupt specific complex formation are designed, it is essential that ligands that associate specifically with individual paralogs are identified. The results in this report and our previous publications (Bose et al., 2021) demonstrate that the mutants that alter the conserved aspartic acid and glutamic acid residues to alanine in the phospho-peptide binding permit the identification of ligands specific for each 14-3-3 paralog, thereby allowing the development of these complexes as potential therapeutic targets.

The results in this report suggest that 14-3-3ε inhibits centrosome function by inhibiting the activity of Plk1 and separase. This is consistent with previous data suggesting that Plk1 and separase cooperate to promote centriole disengagement in human cells (Tsou et al., 2009). Further evidence suggests that phosphorylation of pericentrin by Plk1 results in the increased cleavage of pericentrin by separase leading to disengagement (Kim et al., 2015). In addition to the cleavage of pericentrin, Plk1 also promotes the cleavage of Cep68 in prometaphase, which is followed by the degradation of pericentrin and the release of Cep215 from the centrosome leading to disengagement and licensing (Pagan et al., 2015). These results are consistent with our data, which illustrates that expression of the 14-3-3ε D127A mutant results in an increase in centrosome size, a decrease in the distance between centrioles and increased retention of Cep215 and pericentrin at the centriole. In contrast, expression of the 14-3-3ε E134A mutant results in a decrease in centrosome size, increased distance between centrioles and decreased retention of Cep215 and pericentrin at the centriole. Consistent with these results, we demonstrate that the levels of the activating phosphorylation on Plk1 (T210) are decreased in cells expressing D127A and elevated in cells expressing E134A. Treatment with Plk1 or separase inhibitors exacerbates the phenotypes observed with the D127A mutant and prevents the premature centriole disengagement in interphase observed in cells expressing the E134A mutant. In contrast, expression of Plk1 or separase mutants that do not bind to 14-3-3ε leads to increased disengagement and centriole splitting in cells expressing the E134A mutant. Our results suggest that the overexpression of WT Plk1 results in centriole reduplication, whereas S99A Plk1 expression leads to disengaged centrioles. Plk1 regulates multiple events that govern centrosome duplication, centrosome separation and centriole disengagement. When Plk1 WT is overexpressed, it is active and leads to centrosome amplification via influencing the activity of LKB1, cyclin B–Cdk1and axin (Liu and Erikson, 2002; Ruan et al., 2012; Werle et al., 2014). However, it is possible that the S99A mutant might not disrupt other Plk1 functions, leading to just an increase in disengagement. Furthermore, whereas treatment with both a Plk1 inhibitor and a separase inhibitor prevents premature centriole disengagement, treatment with both inhibitors does not significantly decrease centriole disengagement. However, co-expression of mutants in Plk1 and separase that do not bind to 14-3-3ε results in a significant increase in centriole disengagement and premature centriole cleavage, a result distinct from that observed for the dual inhibitor treatment. These results could be because Plk1 is upstream of separase in the disengagement pathway, and suggest that 14-3-3ε inhibits the activity of both Plk1 and separase to prevent premature disengagement.

In addition to the cleavage of the cohesion complex leading to sister chromatid separation at anaphase, separase is also required for centriole disengagement by stimulating the cleavage of pericentrin (Lee and Rhee, 2012; Matsuo et al., 2012). We have not determined whether any defects in chromatid segregation are observed in these cells. However, previous work from the laboratory has demonstrated that cells lacking 14-3-3ε preferentially undergo multipolar mitoses, whereas cells lacking 14-3-3γ undergo pseudo-bipolar mitoses with lagging chromosomes (Tilwani et al., 2021). We did not observe any defects with chromatid separation in those experiments, though we did observe lagging chromosomes in cells with a knockout of 14-3-3γ, suggesting that the defects we observed are probably due to centrosome dysfunction. Multiple reports have suggested that cleavage of the cohesion complex is synchronized with centriole disengagement to permit the timely completion of mitosis (Nakamura et al., 2009; Schockel et al., 2011; Thein et al., 2007), although other reports have suggested that disengagement might occur before sister chromatid separation (Agircan and Schiebel, 2014). However, all of these reports suggest that premature separase activation can lead to premature disengagement and centriole splitting (Karki et al., 2017; Nakamura et al., 2009; Schockel et al., 2011; Thein et al., 2007). These results are consistent with our observations that expression of the 14-3-3ε E134A mutant, which does not bind effectively to separase, results in centriole disengagement and premature centriole splitting in interphase cells.

In addition to regulating centriole disengagement at the end of mitosis, both Plk1 and separase are required for other functions during mitosis. Plk1-mediated phosphorylation of Cdc6 leads to complex formation between Cdc6 and cyclin B–Cdk1, leading to separase activation (Yim and Erikson, 2010). In addition, Plk1 phosphorylates Cdc25C leading to Cdc25C nuclear transport, activating the cyclin B–Cdk1 complex and mitotic progression (Toyoshima-Morimoto et al., 2002). As 14-3-3ε is required to inhibit Cdc25C function in interphase, preventing the premature activation of the cyclin B–Cdk1 complex (Dalal et al., 2004), it is possible that in addition to binding to and inhibiting Plk1 and separase activity, loss of 14-3-3ε or the expression of the E134A mutant of 14-3-3ε, could indirectly lead to an increase in Plk1 and separase activity leading to premature centriole disengagement. This is consistent with our observation that the 14-3-3ε E134A mutant shows decreased complex formation with Cdc25C and previous reports demonstrating that 14-3-3ε inhibits Cdc25C function (Dalal et al., 2004; Telles et al., 2009). Similarly, expression of the D127A mutant decreases the levels of the activating phosphorylation of Plk1 at T210 by the kinase Aurora A. In contrast, expression of E134A leads to an increase in the levels of phosphorylation of Plk1 on T210. Treatment with a Cdk1 inhibitor does not lead to a decrease in the phenotypes associated with the expression of the E134A mutant, suggesting that the effects of 14-3-3ε on centriole disengagement might be independent of the ability of 14-3-3ε to inhibit Cdc25C function. However, we cannot rule out the likelihood that in addition to the ligands we have identified, 14-3-3ε might have other functions that contribute to these phenotypes.

Previous results have demonstrated that Plk1 forms a complex with 14-3-3γ and 14-3-3ζ (Du et al., 2012; Kasahara et al., 2013). 14-3-3ζ binds to Plk1 phosphorylated at S330 and S597; these residues are distinct from S99, and the 14-3-3ζ Plk1 interaction regulates cytokinesis (Du et al., 2012). Furthermore, the loss of 14-3-3ζ does not lead to centrosome amplification (this report), unlike the loss of 14-3-3ε (Mukhopadhyay et al., 2016; Tilwani et al., 2021). 14-3-3γ binds to Plk1 when it is phosphorylated at S99 (Kasahara et al., 2013), the same residue that we have identified as being required for complex formation with 14-3-3ε (this report) and loss of either 14-3-3γ or 14-3-3ε leads to centrosome amplification (Mukhopadhyay et al., 2016; Tilwani et al., 2021). However, our previous results suggest that 14-3-3γ inhibits centriole duplication by binding to NPM1 and that expression of the 14-3-3γ D129A mutant leads to the formation of a disengaged centrosome that cannot induce centriole duplication (Bose et al., 2021), a phenotype distinct from that reported for 14-3-3ε in this report. These results suggest that the two 14-3-3 paralogs regulate distinct steps in the centrosome duplication pathway.

**Fig. 7.**
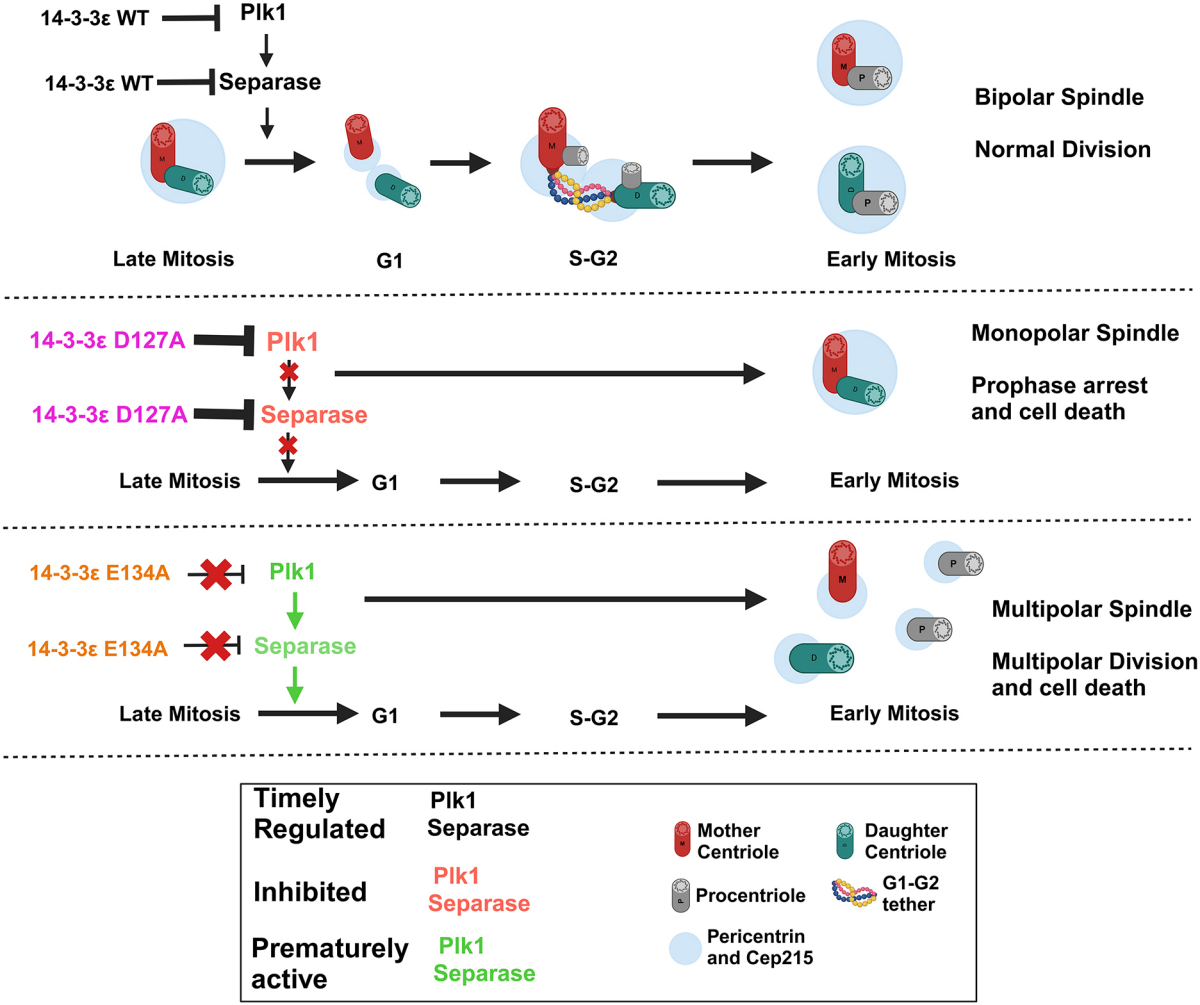
**Model for regulation of centriole disengagement by 14-3-3ε.** 14-3-3ε inhibits premature centriole disengagement by inhibiting the activity of Plk1 and separase. 14-3-3ε mutants showing increased or decreased affinity for Plk1 and separase, inhibiting disengagement or promoting premature centriole disengagement, respectively. Created in BioRender by Dalal, S., 2025. https://BioRender.com/z12e7mt. This figure was sublicensed under CC-BY 4.0 terms.

The results in this report lead to the following model (Fig. 7). 14-3-3ε inhibits the activity of Plk1 and separase in late interphase and part of mitosis, preventing premature centriole disengagement. The 14-3-3ε D127A mutant binds with greater affinity to both Plk1 and separase, inhibiting centriole disengagement through interphase. This leads to monopolar spindle formation in mitosis, resulting in cell death. In contrast, expression of the E134A mutant that shows a decreased affinity for Plk1 and separase leads to premature centriole disengagement and centriole splitting, resulting in the formation of multipolar spindles, which post cytokinesis, results in cell death. In conjunction with our previous studies, these results demonstrate that different 14-3-3 paralogs regulate different stages in the centrosome cycle and argue for paralog-specific regulation of a subset of protein ligands.

## MATERIALS AND METHODS

### Reagents

A list of reagents and suppliers is in Table S1.

### Cell lines and transfections

HCT116 (RRID: CVCL_0291), HEK293 (RRID: CVCL_0045), hTERT-RPE1 (RRID: CVCL_4388) and HeLa Kyoto EGFP-alpha-tubulin/H2B-mCherry (RRID: CVCL_L802) cells were cultured as described previously (Bose et al., 2021; Tilwani et al., 2021). All the cell lines were verified by short tandem repeat (STR) profiling and were mycoplasma free. HCT116 cell-derived 14-3-3ε and 14-3-3ζ knockout lines were generated as described previously (Tilwani et al., 2021) using the guide RNA sequences in Table S2. Transfections were performed with polyethylenimine (PEI) (Polysciences, Inc.) or Lipofectamine 3000 (Invitrogen) as per the manufacturer's instructions. Where indicated, cells were treated with the vehicle control or the following drugs at the indicated concentrations for 20–24 h, followed by immunofluorescence analysis, western blotting and flow cytometry. Drug concentrations are in the Table S1.

### Plasmids

Sequences of the oligonucleotides used in this study are in Table S2. The 14-3-3ε-derived mutant constructs (D127A, E134A and D127AE134A) were generated by site-directed mutagenesis as described previously (Vishal et al., 2018). WT and mutant 14-3-3ε WT constructs were cloned into pCMVmOrange digested with EcoRI and XhoI. The HA-tagged 14-3-3ε WT and mutants (D127A, E134A and D127AE134A) were amplified by PCR and cloned into pCDNA3HA (Bose et al., 2021) digested with BamHI and XhoI. pcDNA5 FRT TO myc hSeparase was Addgene plasmid #59820 (deposited by Stephen Taylor; Holland and Taylor, 2006). The mutants (LD, AD, AC, S1501A and T1363A) were generated using site-directed mutagenesis or amplified in PCRs. We also used pRcCMV myc-Plk1 WT (Addgene plasmid #41160), pRcCMV myc-Plk1 T210D (Addgene plasmid #41158) (Golsteyn et al., 1994) and pEGFP-Centrin2 (Addgene plasmid #41147) (Kleylein-sohn et al., 2007) (deposited by Erich Nigg), and the Plk1 point mutants (S99D, S99E, S99A, and T210A), which were generated site-directed mutagenesis. The14-3-3ε WT and mutants (D127A, E134A and D127AE134A) were cloned into pGEXT-4T-1 using primers shown in Table S2.

### Western blot analysis

Protein extracts were prepared and resolved on SDS-PAGE gels followed by western blotting with antibodies to the indicated proteins as previously described (Bose et al., 2021). The blots were developed with Clarity (Bio-Rad) western blot chemiluminescent substrate (Clarity™ Western ECL Substrate), Femto (Bio-Rad) western blot chemiluminescent substrate or Femtolucent plus HRP chemiluminescent reagents and imaged on a ChemiDoc™ Imaging System (Bio-Rad). Antibody dilutions are in Table S3. Uncropped images of western blots from this paper are shown in Fig. S8.

### Immunofluorescence assays

Immunofluorescence assays (IFAs) were performed as previously described (Bose et al., 2021). Briefly, post-transfection, the cells were synchronized or treated with inhibitors as described above and fixed and stained with the indicated antibodies (Table S1). Confocal images were acquired on an LSM 780 Carl Zeiss confocal microscope or Leica SP8 confocal microscope using either 63× oil or 100× oil objective lens with 5×–6× optical zoom. An image of the entire cell was acquired with 0.35 μm sections. Acquired images were processed using the LSM Image Browser or LASX software, where the image was represented in 2D by the projection of the entire *z*-stacks. The insets were derived from the Extract Region tool in the LSM Image Browser. The quantification of intensity of Cep215, pericentrin, the area of centrosome and the distance between the centriole was calculated in a minimum of 30 cells per set for CEP215 and a minimum of 70 cells per set for pericentrin in three independent experiments using Image J FIJI software. For determining the number of centrosomes, the spot ratios for pericentrin (PCNT)/centrin (CNT; GFP–Centrin2), rootletin/CNT, Cep68/CNT, ODF2/CNT, γ-tubulin/CEP110 and CEP110/SAS6 for 100 cells per set and for rootletin/CEP110 for 50 cells per set of cells expressing the desired constructs were manually counted either on an LSM 780 Carl Zeiss confocal microscope or Leica SP8 confocal microscope or Olympus Inverted IX73 fluorescence or Zeiss AXIO Imager.Z1 upright fluorescence microscope in three independent experiments. Antibody dilutions are in Table S3.

### Proximity ligation assays

HCT116 cells were cultured in eight-well chambered slides at a confluency of ∼50%, transfected with the respective constructs and fixed with 4% paraformaldehyde (PFA) for 20 min and then PLAs were performed using Duolink^®^ PLA Reagent by following the Duolink^®^ PLA Fluorescence protocol. Briefly, fixed cells were permeabilized (using 0.3% Triton X-100 and 0.1% NP-40), blocked (using Blocking solution) and then incubated with the respective primary antibody dilution (using Antibody Diluent) as mentioned in Table S1 for either 2 h at room temperature (RT) or overnight at 4°C. The cells were washed three times with 1× wash buffer 1 and incubated with PLA Probes (PLUS and MINUS) followed by Duolink® Fluorescence Detection Reagent (red or far red) which includes ligation and amplification. The cells were washed twice with 1× wash buffer post ligation and amplification followed by a final wash with 0.01× Wash Buffer B for 1 min. Cells were then mounted using 10 μl of mounting medium with DAPI, and images were acquired using an LSM 780 Carl Zeiss confocal microscope using either a 40× oil or 63× oil objective with 4–6× optical zoom, and images were processed as described above. *Z*-stack-projected images were analyzed using the Image J FIJI software to determine the average number of PLA puncta per cell. The total number of puncta (red) per field was divided by the total number of cells per field (DAPI, blue) to get the average number of puncta per cell. The mean and standard deviation of three independent experiments are plotted.

### Electron microscopy

HCT116 cells stably expressing vector control, and HA-tagged 14-3-3ε WT and mutants (D127A and E134A) were synchronized in S–G2 with RO3306 for 18 h, trypsinized, and processed as described previously (Tilwani et al., 2021). The grids were imaged on a JEM 1400 PLUS transmission electron microscope. Images were analyzed using iTEM software (OSIS) and Image J FIJI software to calculate the centriole length, inner diameter and outer diameter. To calculate the length of the centriole, an average of three vertical microtubule lengths was calculated for each centriole. To determine the inner diameter, the average of the three inner diameters of the centriole ring formed by connecting the first tubule of the triplet for each centriole was calculated. To determine the outer diameter, the average of the three outer diameters of the centriole ring formed by connecting the third tubule of the triplet for each centriole was calculated. The minimum number of cells analyzed to determine the length of the centriole was *N*≥15, and for determining inner and outer diameter, *N*≥30 in three independent experiments.

### Live-cell imaging, proliferation assays and flow cytometry

HeLa KYOTO cells were transfected with HA pcDNA3 vector control, HA-tagged 14-3-3ε WT, and HA-tagged 14-3-3ε (D127A and E134A), and stable cells were generated after selection with G418. Post selection, the expression was confirmed by western blotting, and the cells were subjected to live-cell imaging on anOlympus 3i spinning disc microscope. For the inhibitor experiments, the cells were treated with vehicle (DMSO), 200 nM BI2536 or 10 μM Sepin1 or both, and the images were acquired using 60× oil immersion objective lens with 488 and 568 excitation wavelengths, in the following condition: 37°C, 5% CO_2_ and 75% humidity. The imaging was initiated by locating the cells in late G2 where two microtubule-organizing centers were visible, or in prophase. 15 fields were selected for sequential imaging in each experiment with an image being acquired at intervals of 15-20 min. Once the *Z*-stack of a given field was acquired (1–1.5 min), acquisition of the next field began until all fields were acquired for a particular time point. The images of the same cell were acquired at an interval of 15–20 min over 20–24 h. The video and images were processed using Slide Book 6 software, showing the time stamp at the top and the scale at the bottom.

HCT116 cells cultured in glass bottom 24 well plates were transfected with vector control, mOrange tagged 14-3-3ε WT and mutant (D127A and E134A) constructs. 24 h post-transfection, proliferation was measured by acquiring the images using 568 nm excitation wavelength and phase contrast of the entire well at intervals of 1 h over 48 h, for each construct, on a Sartorius Incucyte live-cell imaging system using a 20× objective. The cells were maintained at 37°C, 5% CO_2_ and 70% humidity during acquisition. The data was analyzed by training the Incucyte AI software by selecting random fields to sense the number of mOrange-positive cells and the total number of cells (using phase contrast). The fold change was determined by dividing the total number of mOrange-positive cells per field by the initial number of mOrange-positive cells (phase contrast) per field for each time point and plotted.

Trypsinized HCT116 cells treated with the drugs were washed with PBS, fixed with ice-cold 70% ethanol and then incubated with 0.1 mg/ml propidium iodide and RNase for 30 min. The samples were run on a Thermo Fisher Attune NxT Flow Cytometer, and data analysis and graph were obtained using Modfit software.

### GST pulldown and co-immunoprecipitation assays

GST pulldown and co-immunoprecipitation assays were performed as described previously (Bose et al., 2021). HCT116 cells were grown up to 70% confluency and were transfected with either Myc tag Plk1 (WT, S99A, S99D, S99E and T210A) constructs or Myc-tagged h-separase (WT, S1501A and T1363A) constructs. At 48 h post-transfection, cells were lysed in EBC lysis buffer [0.5 mM Tris-HCl pH 8.0, 125 mM NaCl, 0.5% (v/v) NP-40, protease inhibitor cocktail, phosphatase inhibitor cocktail]. Following centrifugation (13,000 ***g*** for 20 min), 10% input was removed, and lysates were incubated with an equal amount of GST only, 14-3-3εWT or 14-3-3ε mutants (D127A, 14-3-3εE134A, 14-3-3εD127AE134A) GST fusion proteins bound to glutathione beads for either 4–5 h at RT or 16 h at 4°C. Post incubation, the reactions were washed three times with NET-N [1 M Tris-HCl pH 8.0, 2.5 M NaCl, 0.5 M EDTA pH 8.0, 0.5% (v/v) NP-40] and the complexes resolved on 10% SDS-PAGE gels followed by western blotting.

For endogenous immunoprecipitation, near-confluent HCT116 cells were lysed in EBC buffer, 10% input was removed and the remaining supernatant was divided into two; one portion was incubated with 14-3-3ε or Plk1 or separase the other with rabbit IgG. After 3 h of incubation, 50% slurry of protein G–Sepharose beads was added for 1 h at 4°C and then three washes with 50 mM NET-N were given. The complex was resolved on 10% SDS-PAGE gels followed by western blotting.

For co-immunoprecipitation, 70% confluent HCT116 cells were transfected with HA vector control, HA-tagged 14-3-3ε (WT, D127A, E134A and D127AE134A) along with Myc tag Plk1 (WT, S99A, S99D, S99E and T210A) constructs. Lysates were prepared and incubated for 3 h at 4°C with anti-HA antibody (12CA5) to precipitate HA-tagged proteins. The reaction was incubated with protein G–Sepharose beads for 1 h at 4°C and washed three times with NET-N. The reactions were resolved on 10% SDS-PAGE gels followed by western blotting.

### CD spectra

pGEX-4T-1 14-3-3ε WT and mutant constructs were purified as described previously (Bose et al., 2021). The recombinant proteins were eluted with 10 mM reduced glutathione and dialyzed against with 20 mM Tris-HCl pH 8, NaCl 20 mM overnight. The proteins were concentrated to 15-20 μM, and CD spectra was performed on the Jasco J-1500 CD Spectrophotometer, and the data was processed in Jasco software.

## Supplementary Material

10.1242/joces.263808_sup1 Supplementary information
