# Supplementary material for: 14-3-3ε inhibits premature centriole disengagement by inhibiting the activity of Plk1 and separase
Source: J Cell Sci. 2025 Jul 18;138(14):jcs263808. doi: 10.1242/jcs.263808 (PMC12301659; doi:10.1242/jcs.263808)
Supplement: Supplementary information [file joces-138-263808-s1.pdf]

SUPPLEMENTARY FIGURE 1

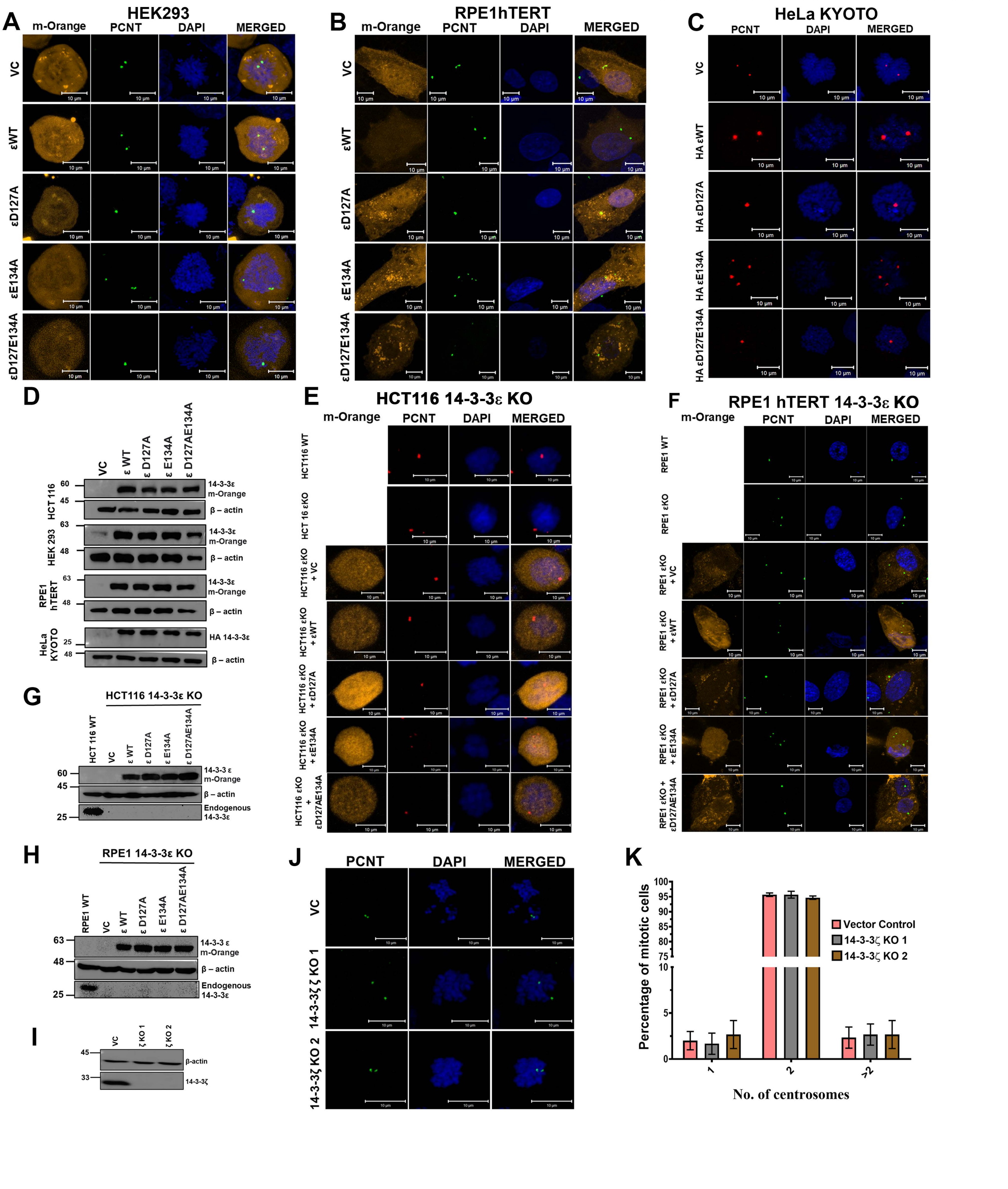

Journal of Cell Science • Supplementary information

Supplementary Figure 1. 14-3-3ε mutants alter centrosome numbers in different cell lines. (A-C) Representative images of the indicated cell lines transfected with the mOrange tagged wild type (WT) and mutant (D127A, E134A, D127AE134A)14-3-3ε constructs. The quantitation is in Figure 1C-E. **(D)** Western blot analysis showing equal expression of the different 14-3-3ε constructs in the different cell lines. β-actin served as a loading control. **(E-H)** HCT116 (E) and RPE1hTERTTert (F) derived 14-3-3ε knockout lines transfected with the indicated constructs were stained with antibodies to pericentrin, and representative images are shown. The quantitation is in Figure 1F-G. Protein extracts from the transfected cells were resolved on SDS-PAGE gels, followed by Western blotting with the indicated antibodies(G-H). β-actin served as a loading control. **(I-K)** HCT116 derived 14-3-3ζ knockout cells were generated. Protein extracts prepared from the vector control and 14-3-3ζ knockout cells were resolved on SDS PAGE gels, followed by Western blotting with the indicated antibodies (I). Note that the knockout cells have low levels of 14-3-3ζ. The vector control and knockout cells were arrested in mitosis, stained with antibodies to pericentrin, and counterstained with DAPI. Representative images are shown (J), and the mean and standard deviation of three independent experiments are plotted (K). p-values were obtained using unpaired student's t-test with Welch's correction. ns – non-significant, $*p < 0.05$. Scale= 10μm.

Journal of Cell Science • Supplementary information

SUPPLEMENTARY FIGURE 2

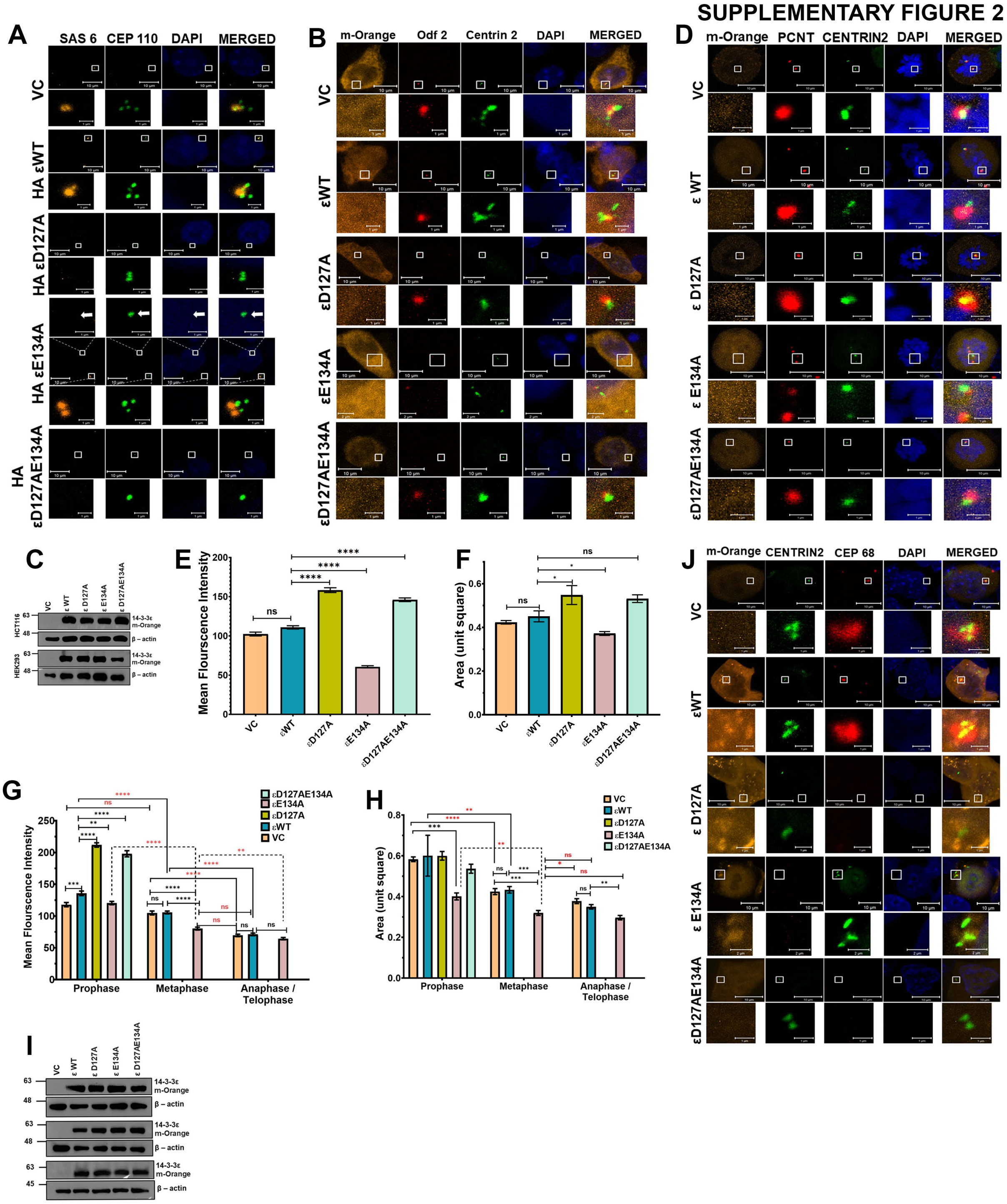

Journal of Cell Science • Supplementary information

Fig. S2. 14-3-3ε prevents centriole disengagement. (A) HCT116 cells stably expressing the indicated HA-tagged constructs were stained with antibodies to SAS6 and CEP110. Representative images are shown. White arrows indicate disengaged single CEP110 focus not associated with SAS6 (representative of 1CEP110 / 0SAS6 ratio). (B) HCT116 cells co-transfected with the indicated mOrange tagged 14-3-3ε constructs and GFPCentrin2 were stained with antibodies to ODF2. Representative images are shown. **(C)** Western blot showing the expression of mOrange tagged 14-3-3ε constructs (WT, D127A, E134A, D127AE134A) for experiments shown in figure 2D-F. β-actin served as a loading control. **(D)** Representative images for the experiment are shown in Figure 2F). **(E-H)** For experiments shown in figure 2D-E, we determined the intensity of pericentrin staining (E) and centrosome area as measured by pericentrin staining (F). In three independent experiments, the intensity of pericentrin staining (G) and centrosome size (H) were measured in 30 cells in the indicated mitotic stages. The mean and standard error of three independent experiments are plotted. p-values were obtained using unpaired student's t-test with Welch's correction for (E-F) and one-way ANOVA (Tukey's multiple comparison) for (G-H). **(I)** Western blot showing the expression of mOrange tagged 14-3-3ε constructs (WT, D127A, E134A, D127AE134A) for experiments shown in figure 2F-J. Top panel for figure (2F-G), middle panel for figure (2H) and SF2J and bottom panel for figure (2I-J) and Supplementary figure 2H. β-actin served as a loading control. **(J)** Representative images for the experiment in figure 2G are shown.

Journal of Cell Science • Supplementary information

SUPPLEMENTARY FIGURE 3

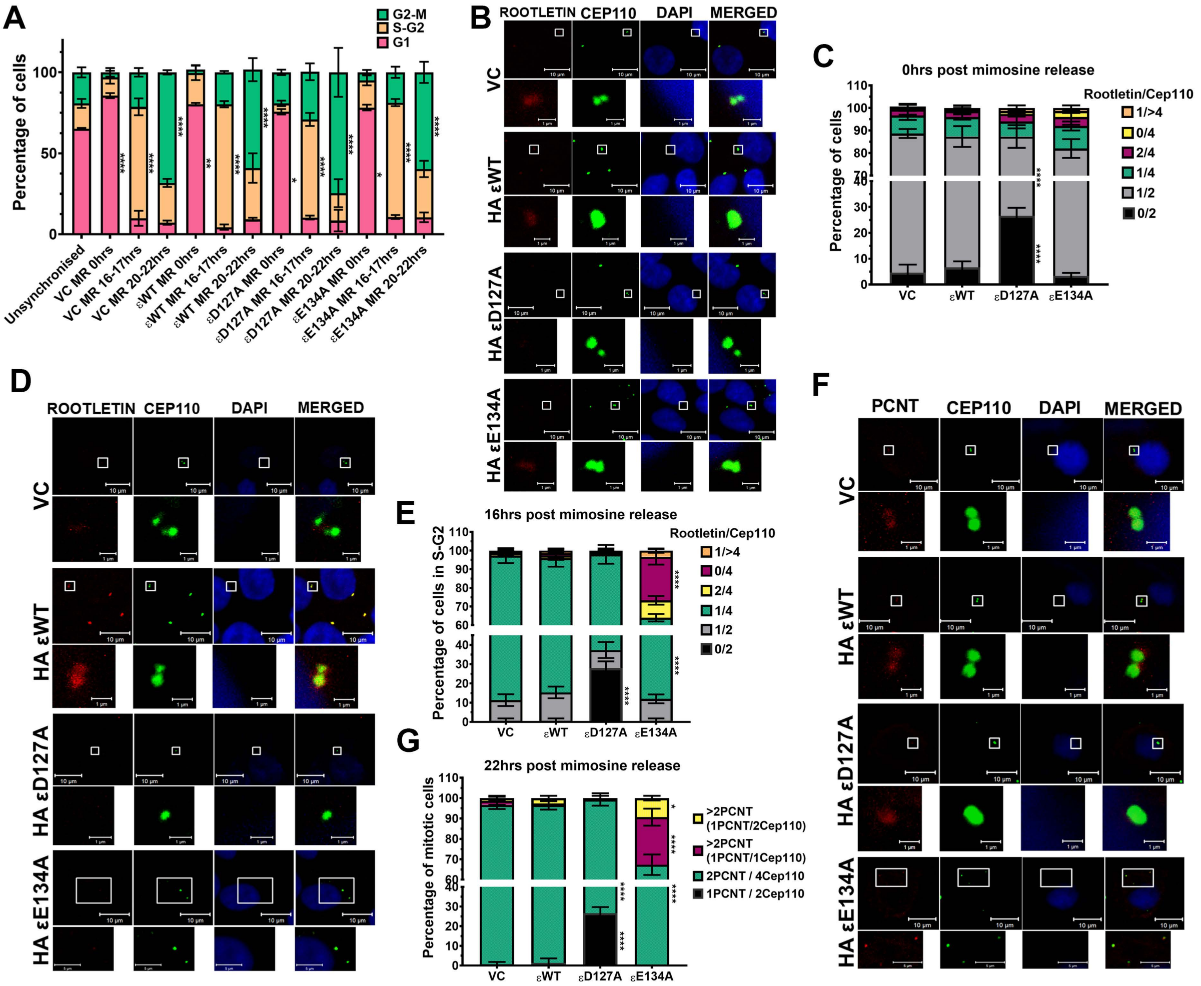

Fig. S3. The expression of the E134A mutant of 14-3-3ε leads to premature centriole disengagement in G2 phase. (A-G) HCT116 cells stably expressing the vector control (VC) or WT and 14-3-3ε mutant constructs were treated with mimosine for 20 hours. Mimosine was removed, and cells were either harvested or allowed to progress through the cell cycle at the indicated times. The cells were harvested and processed for flow cytometry to generate cell cycle profiles. The mean and standard deviation of cells in different phases of the cell cycle in three independent experiments is plotted (A). The cells were stained with antibodies to rootletin and (B-E) and CEP110 and pericentrin (F-G). The rootletin to CEP110 ratio was determined in G1 cells (B-C) and in S-G2 cells (D-E) and the pericentrin to CEP110 ratio in G2/M cells (F-G). Representative images are shown (B, D and F) and the mean and standard deviation of three independent experiments is plotted (C, E and G). p-values were obtained using 2-way ANOVA (Tukey's multiple comparison). *p <0.05, **p < 0.01, ***p < 0.001, ****p <0.0001. Scale = 10μm, inset scale = 1μm or 5μm.

Journal of Cell Science • Supplementary information

SUPPLEMENTARY FIGURE 4

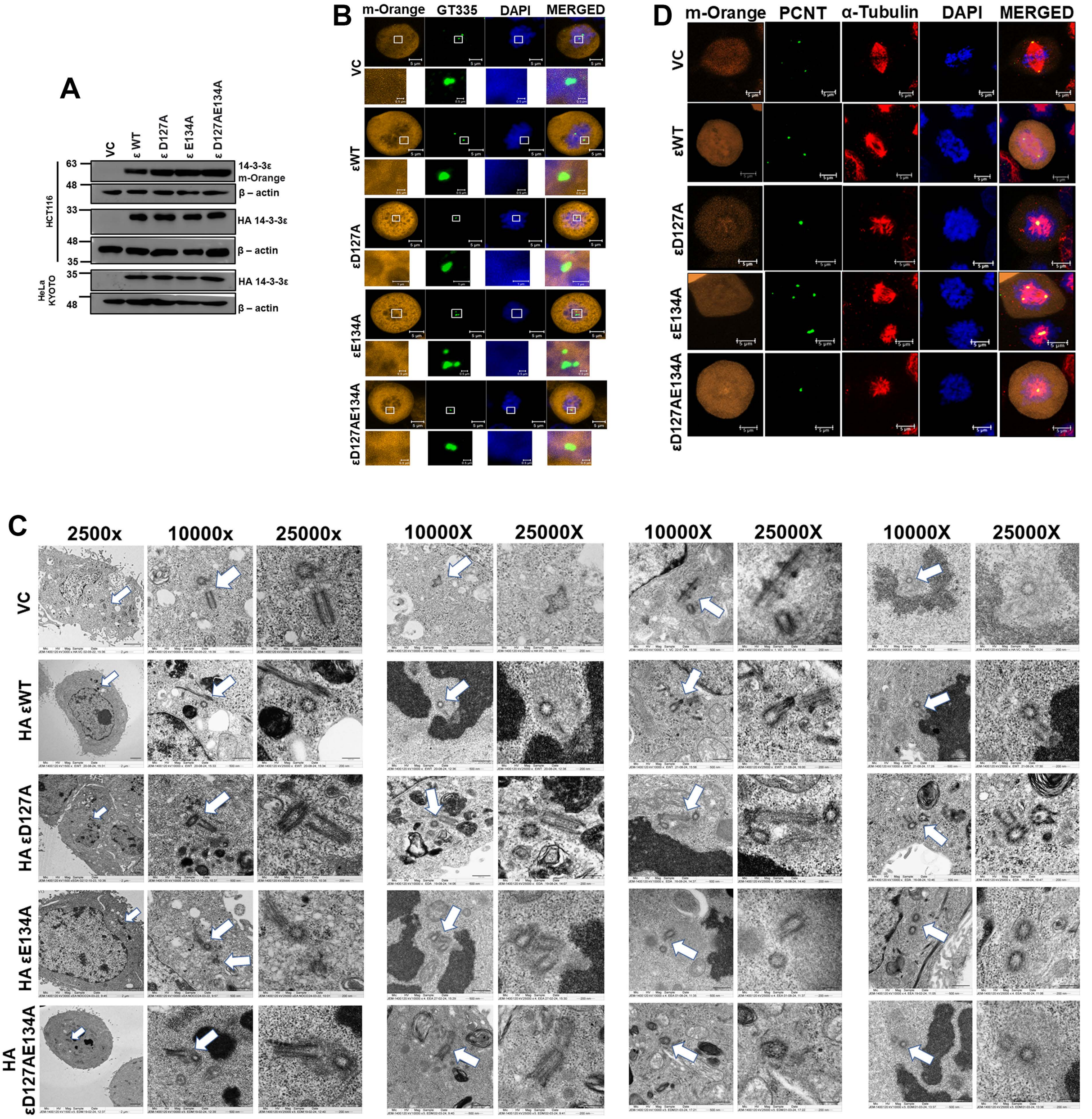

Fig. S4. Expression of the 14-3-3ε mutants leads to a mitotic delay and cell death. (A) Representative blot shows the ectopic expression of mOrange tagged, and HA-tagged 14-3-3ε constructs (WT, D127A, E134A and D127AE134A) in HCT116 cells and HeLa KYOTO for experiments shown in figure 3. β-actin served as a loading control. **(B)** HCT116 cells transfected with the indicated constructs were stained with antibodies to glutamylated tubulin (GT335 green) and counter-stained with DAPI (blue). Scale = 5μm, inset scale = 0.5μm. **(C)** Electron micrographs showing centriole structure indicated with a white arrow in four different cells for each stably expressing HA vector control, and HA-tagged 14-3-3ε constructs (WT, D127A, EI134A and DI27AE134A). Scale= 2μm, 500nm, 200nm. **(D)** HCT116 cells expressing mOrange vector control and mOrange tagged 14-3-3ε constructs (WT, D127A, E134A, D127AE134A) were synchronized in mitosis using nocodazole followed by washing out of the nocodazole allowing mitotic progression. Cells were fixed and stained for Pericentrin (green), α-tubulin (red) and counter-stained with DAPI (blue). Scale= 5μm.

SUPPLEMENTARY FIGURE 5

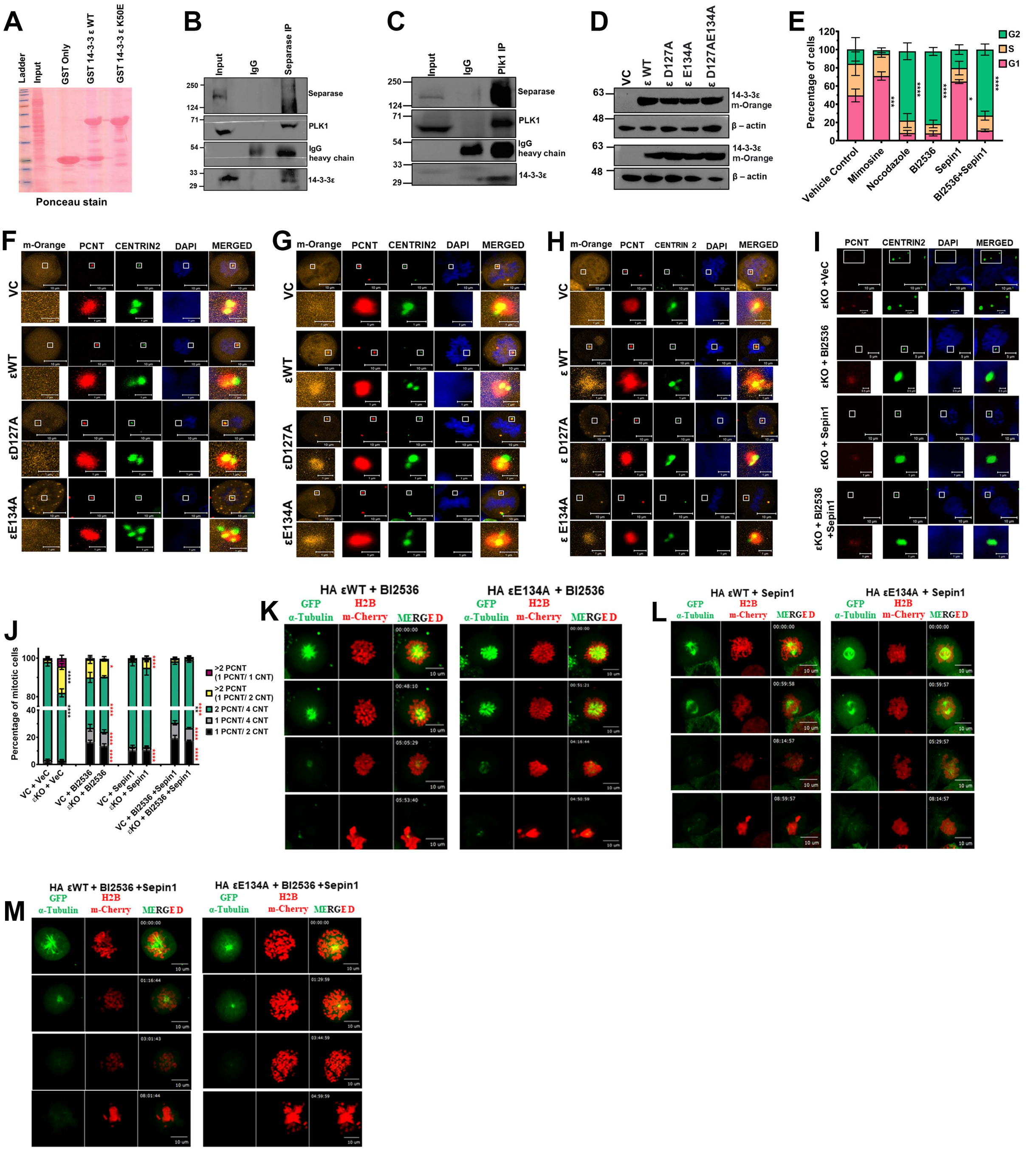

Journal of Cell Science • Supplementary information

Fig. S5. 14-3-3ε forms a complex with Plk1 and Separase. **(A)** Ponceau image of the GST pulldown blot showing band for GST only and GST 14-3- 3ε WT and GST 14-3-3ε K50E. **(B-C)** Protein extracts from the HCT116 cells were incubated with antibodies to Separase (B) or Plk1 (C), and the reactions resolved on SDS PAGE gels followed by Western blotting with the indicated antibodies. Immunoprecipitations with IgG served as negative controls. **(D)** Protein extracts prepared from HCT116 cells transfected with the indicated constructs and treated with BI2536 or Sepin-1 were resolved on SDS PAGE gels, followed by Western blotting with the indicated antibodies. β-actin served as a loading control. **(E)** The cell cycle distribution of the indicated cells treated with either BI2536 or Sepin-1. The vehicle control, mimosine and nocodazole-treated cells served as controls. **(F-H)** HCT116 cells stably expressing GFPCentrin2 were transfected with mOrange vector control and mOrange tagged 14-3-3ε constructs (WT, D127A, E134A) and treated with BI2536, Sepin1 or both (BI2536 and Sepin1) and stained for pericentrin and counterstained with DAPI. Representative images of cells treated with BI2536 (F) with Sepin-1 are shown in (G), and both BI2536 and Sepin-1 (H) are shown. **(I-J)** HCT116 14-3-3ε KO cells treated with Vehicle control (DMSO), BI2536, Sepin1 or both (BI2536 and Sepin1) were stained using antibody for pericentrin, Centrin2 and counterstained with DAPI representative images are shown (I) and PCNT/Centrin2 was determined(J). Mean and standard deviation was plotted and p-values were obtained using 2-way ANOVA (Tukey's multiple comparison). (*(black) comparison within the group with respective vector control treated with drug, *(red) comparison across the group with 14-3-3εKO Vehicle control) *$p < 0.05$, **$p < 0.01$, ***$p < 0.001$, ****$p < 0.0001$. Scale = 10µm, inset scale = 1µm. **(K-M)** HeLa KYOTO cells stably expressing HA 14-3-3ε WT or HA 14-3-3ε E134A were treated with vehicle control (DMSO), BI2536, Sepin-1 or both BI2536 and Sepin-1 and were subjected to live cell imaging at intervals of 15 mins. Representative images of the same cells treated with BI2536 (K), Sepin-1 (L) or both BI2536 and Sepin1(M) are shown, and the time stamp is present in the top left corner and the scale is represented in the bottom right. Scale = 10µm.

SUPPLEMENTARY FIGURE 6

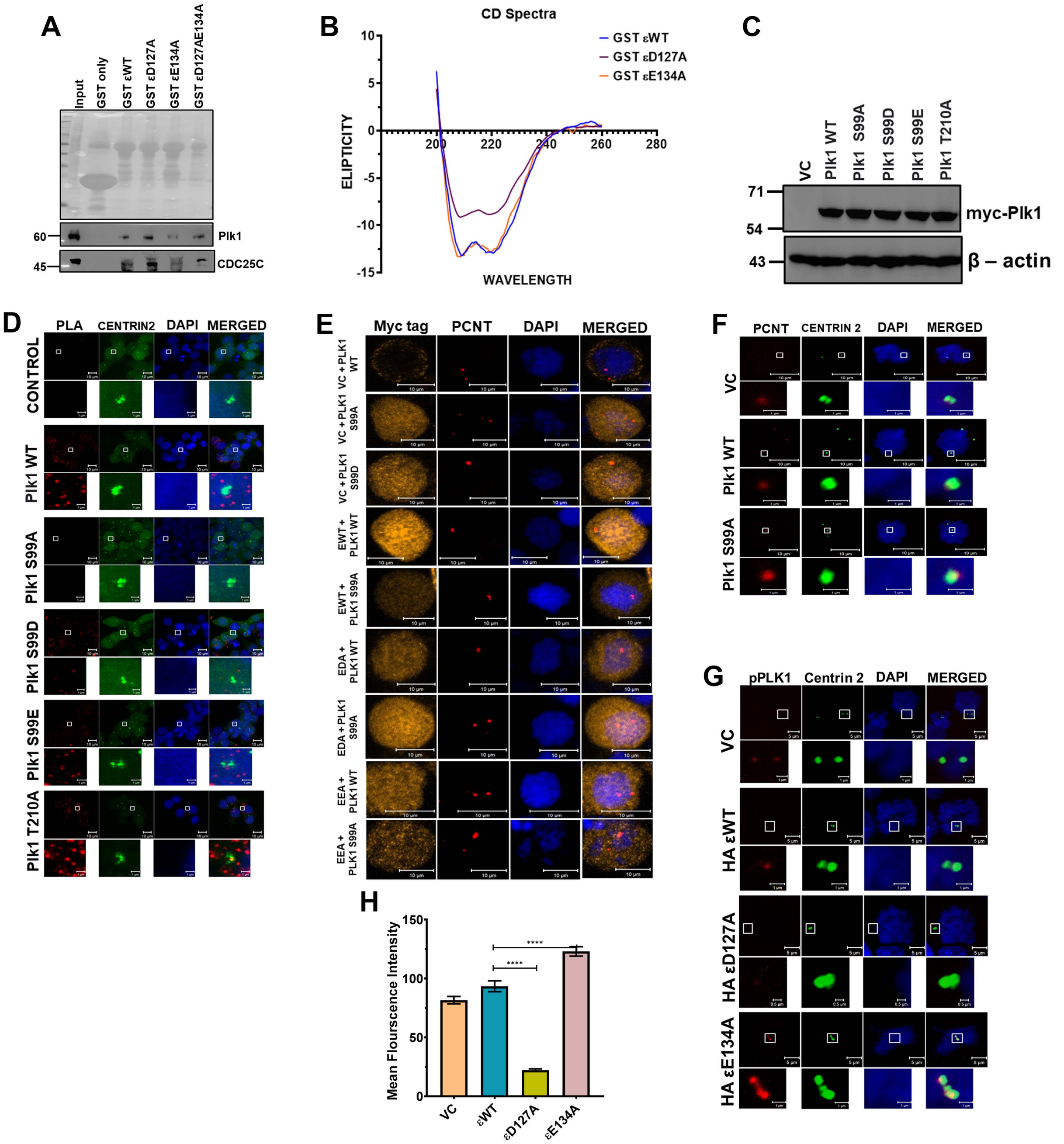

Journal of Cell Science • Supplementary information

Fig. S6. 14-3-3ε inhibits Plk1, preventing centriole disengagement. (A) Protein extracts prepared from HCT116 cells were incubated with GST alone, or GST-tagged WT and mutant (D127A, E134A and D127AE134A) 14-3-3ε fusion proteins immobilized on glutathione Sepharose beads. The reactions were resolved on SDS PAGE gels, and Western blots were performed with the indicated antibodies. GST only served as a negative control, and cdc25C served as a positive control to study the interaction of 14-3-3ε with Plk1. **(B)** A Circular Dichroism (CD) analysis was performed to determine the secondary structure of WT and mutant (D127A and E134A) 14-3-3ε GST fusion proteins. The spectra for the individual proteins are shown. **(C-D)** HCT116 cells stably expressing the vector control or myc-tagged WT and mutant (S99A, S99D, S99E and T210A) Plk1 constructs were to perform PLA assays. Representative images are shown (C), and a Western blot demonstrates that the Plk1 proteins were present at equivalent levels (D). β-actin served as a loading control. **(E)** HCT116 cells stably expressing vector control and HA-tagged 14-3-3ε (WT, D127A and E134A) constructs were transfected with myc-tagged WT and mutant (S99A, S99D and S99E) Plk1constructs, and immunofluorescence staining was performed using antibodies to the myc-epitope tag and pericentrin and the representative images are shown (myc-tag (orange), pericentrin (red) and DAPI (blue)). **(F)** HCT116 cells stably expressing myc-tagged Plk1 WT and Plk1 S99A were arrested in mitosis and stained with antibodies to Centrin2 and pericentrin and counterstained with DAPI. Representative images are shown. **(G-H)** HCT116 cells stably expressing HA vector control, and HA-tagged 14-3-3ε constructs (WT, D127A, EI134A and DI27AE134A) were stained using antibodies against phospho-Plk1-T210, Centrin2 and counterstained with DAPI, representative images are shown in (G). The intensity of the phosphor-Plk1 signal at the centrosome was determined, and mean and standard error were plotted (H). p-values were obtained using unpaired student's t-test with Welch's correction Scale = 10μm and = 1μm in the inset.

Journal of Cell Science • Supplementary information

SUPPLEMENTARY FIGURE 7

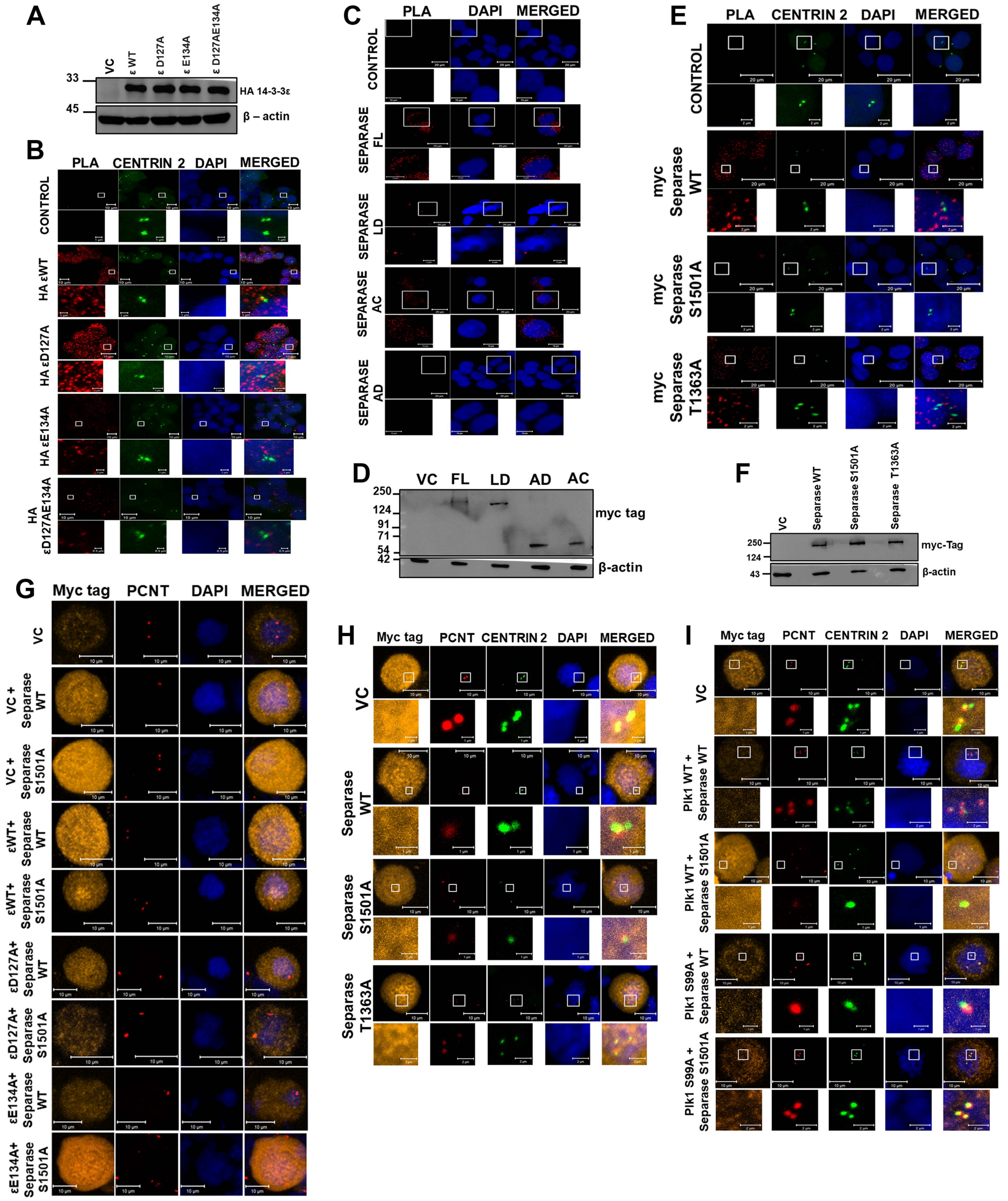

Journal of Cell Science • Supplementary information

Fig. S7. 14-3-3ε inhibits Separase function, inhibiting centriole disengagement. **(A-B)** HCT116 cells stably expressing GFPCentrin2 were transfected with the vector control or HA-tagged WT and mutant (D127A, E134A and D127AE134A) 14-3-3ε constructs and PLA assays were performed. A Western blot analysis demonstrates that the constructs were expressed at similar levels (A). Representative images are shown in (B). Scale = 10µm and inset scale = 0.5µm. **(C-D)** HCT116 cells were transfected with different domains of myc-tagged full-length Separase (FL) and myc-tagged deletion mutants of Separase (Large domain (LD), Auto-catalytic domain (AC), and Active domain (AD)) and PLA assays performed with antibodies to 14-3-3ε and the myc-epitope tag. Representative images are shown in (C). Scale = 20µm and inset scale = 5µm. All Separase mutant constructs were expressed at equivalent levels, and Western blots for β-actin served as loading controls (D). **(E-F)** HCT116 cells stably expressing GFPCentrin2 were transfected with myc-tagged WT and mutant (S1501A and T1363A) Separase constructs and PLA assays performed with antibodies to 14-3-3ε and the myc-epitope tag. Representative images are shown in (E), and a Western blot analysis demonstrates that the proteins were present at similar levels (F). Scale = 20µm and inset scale = 2µm. **(G)** HCT116 cells stably expressing vector control and HA-tagged 14-3-3ε (WT, D127A, E134A) constructs were transfected with myc-tagged Separase WT and myc-tagged Separase (S1501A and T1363A) constructs and immunofluorescence staining was performed using antibodies against myc and pericentrin. Representative images are shown in (G). Scale = 10µm. **(H)** HCT116 cells stably expressing GFPCentrin2 were transfected with myc-tagged Separase WT and myc-tagged Separase mutants (S1501A and T1363A) and stained with antibodies against myc (orange) and pericentrin (red) and counterstained with DAPI (blue). Representative images are shown (H). Scale = 10µm and inset scale = 1µm. **(I)** HCT116 cells stably expressing myc-tagged Plk1 WT and myc-tagged Plk1 mutants S99A were transfected with myc-tagged Separase WT and myc-tagged Separase S1501A and stained with antibodies against pericentrin (red) and Centrin2 (green) and counterstained with DAPI (blue). Scale = 10µm and inset scale = 1µm.

Journal of Cell Science • Supplementary information

BLOT TRANSPARENCY 1

Image in the manuscript

Supplementary 1D

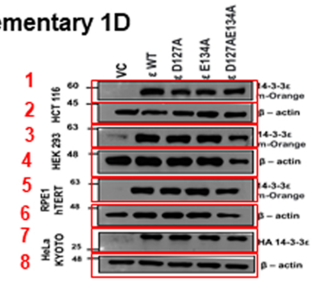

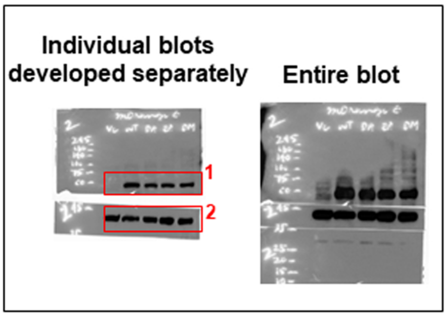

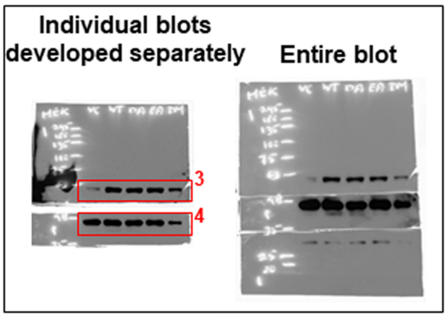

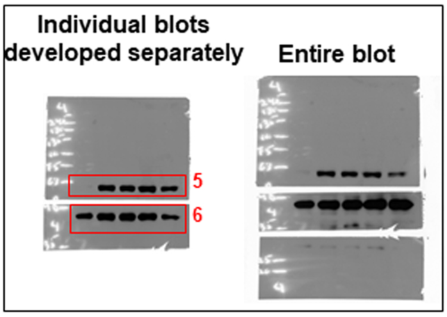

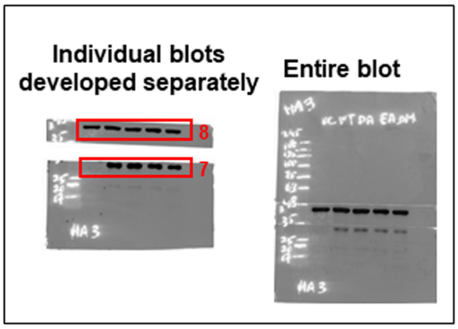

Image in the manuscript

Supplementary 1G

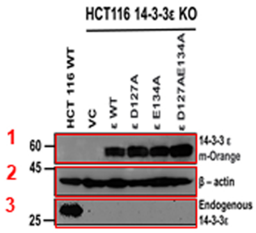

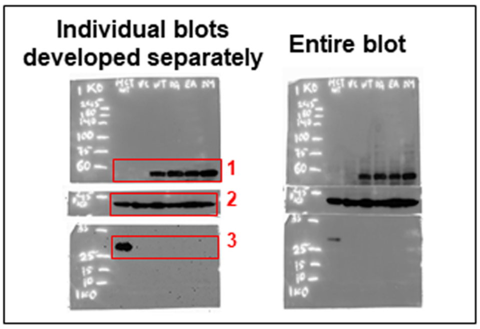

Note: Individually developed blots were used in the manuscript.

BLOT TRANSPARENCY 2

Image in the manuscript

Supplementary 1H

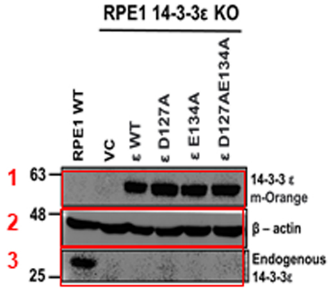

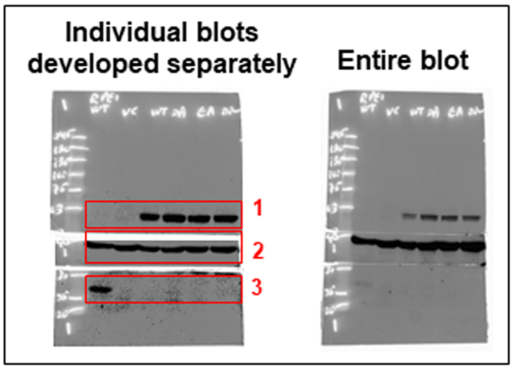

Image in the manuscript

Supplementary 1I

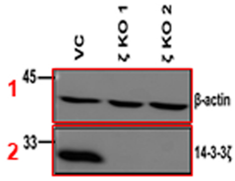

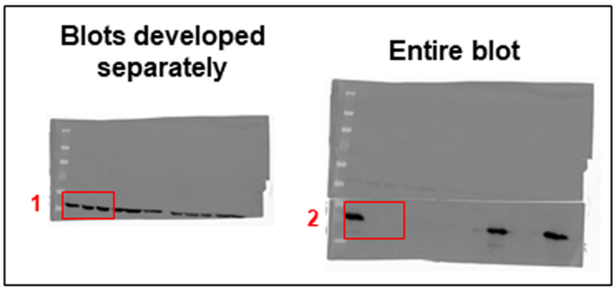

Note: Individually developed blots were used in the manuscript.

BLOT TRANSPARENCY 3

Image in the manuscript

Supplementary 2C

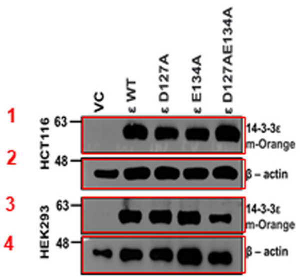

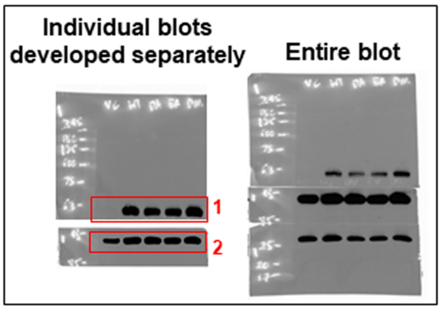

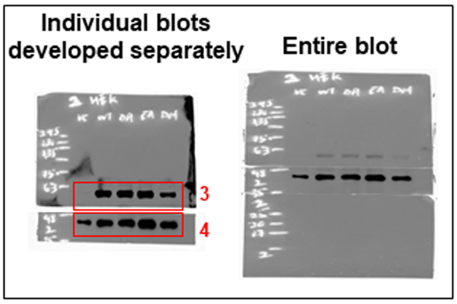

Image in the manuscript

Supplementary 2I

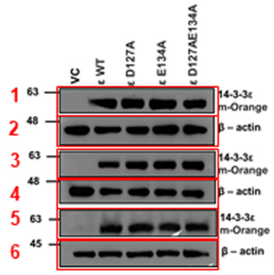

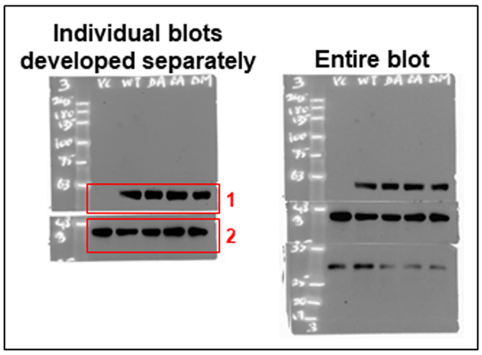

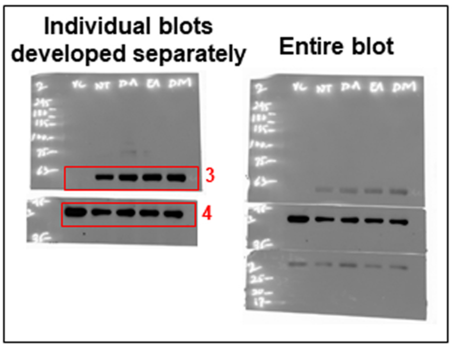

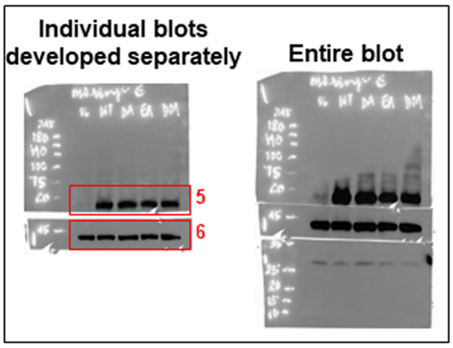

Note: Individually developed blots were used in the manuscript.

Journal of Cell Science • Supplementary information

BLOT TRANSPARENCY 4

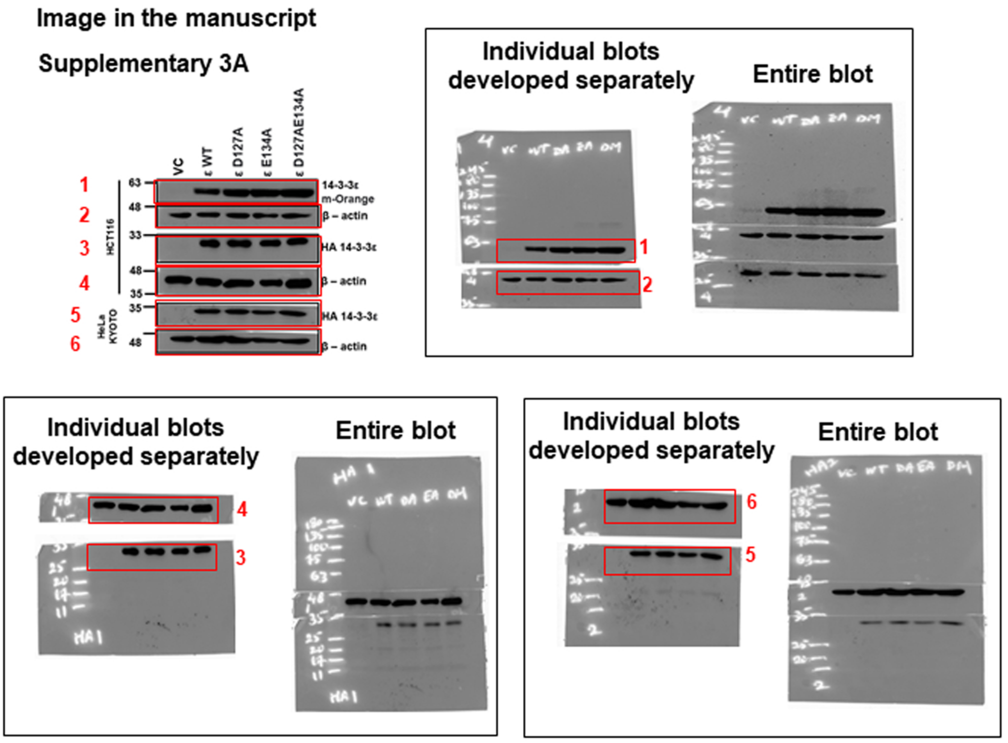

Note: Individually developed blots were used in the manuscript.

Journal of Cell Science • Supplementary information

BLOT TRANSPARENCY 5

Image in the manuscript

FIGURE 4A

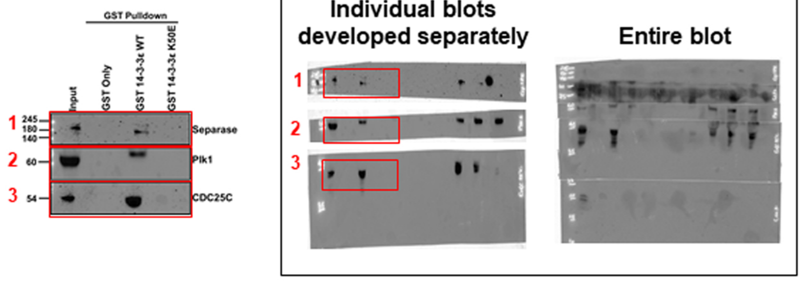

Image in the manuscript

FIGURE 4B

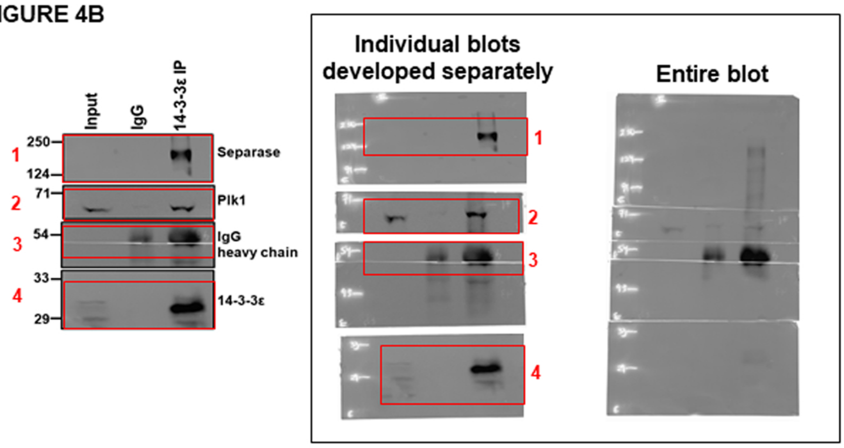

Note: Individually developed blots were used in the manuscript.

Journal of Cell Science • Supplementary information

BLOT TRANSPARENCY 6

Image in the manuscript

Supplementary 5B

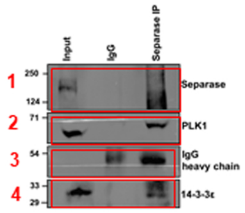

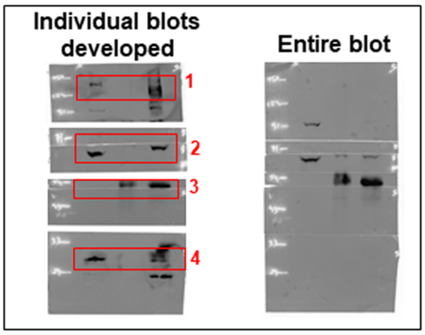

Image in the manuscript

Supplementary 5C

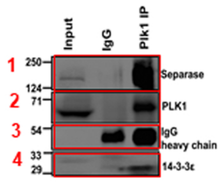

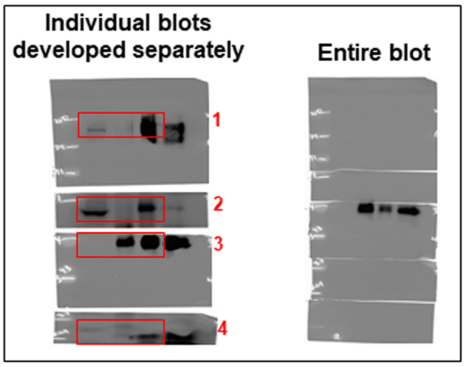

Image in the manuscript

Supplementary 5D

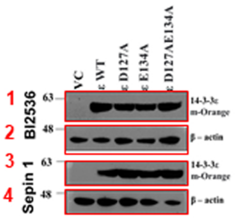

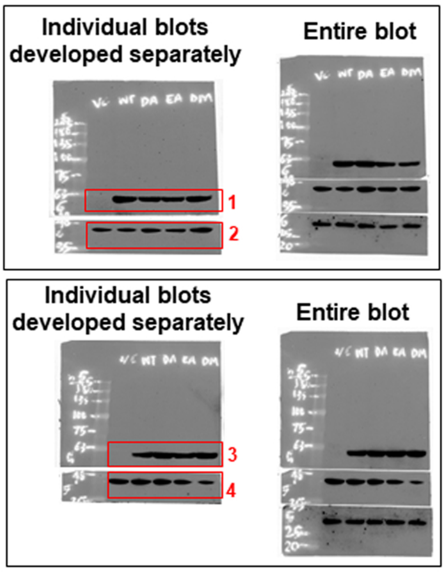

Note: Individually developed blots were used in the manuscript.

Journal of Cell Science • Supplementary information

BLOT TRANSPARENCY 7

Image in the manuscript

FIGURE 5A

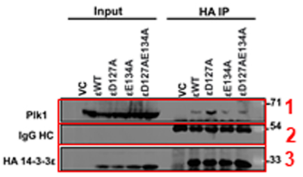

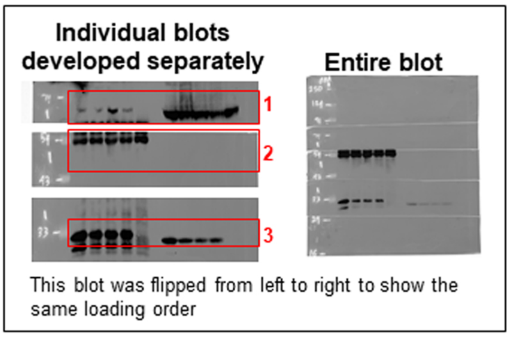

Image in the manuscript

FIGURE 5D

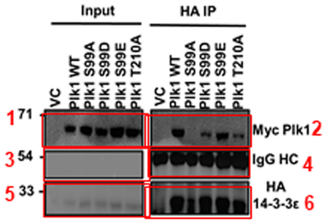

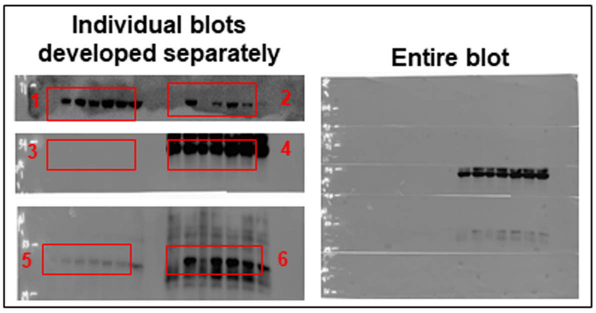

Note: Individually developed blots were used in the manuscript.

Journal of Cell Science • Supplementary information

BLOT TRANSPARENCY 8

Image in the manuscript
Supplementary 6A

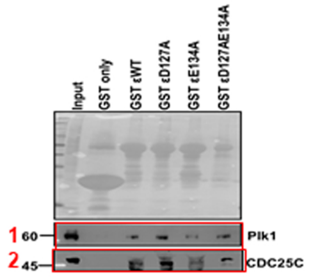

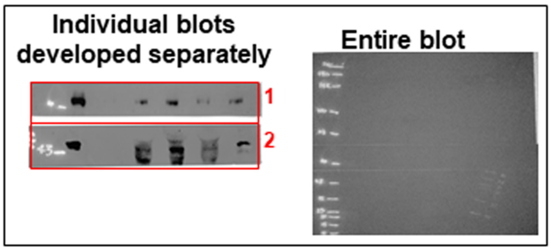

Image in the manuscript
Supplementary 6D

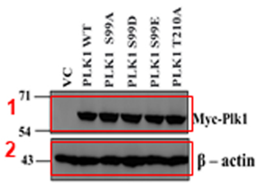

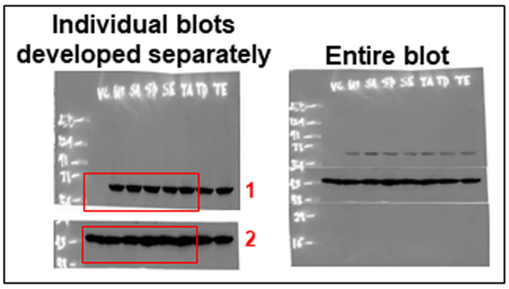

Note: Individually developed blots were used in the manuscript.

Journal of Cell Science • Supplementary information

BLOT TRANSPARENCY 9

Image in the manuscript
Figure 6A

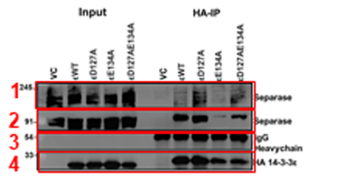

Individual blots developed separately

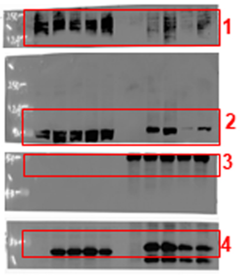

Entire blot

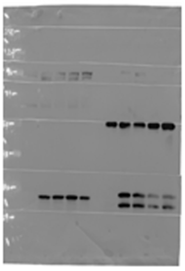

Image in the manuscript

Figure 6F

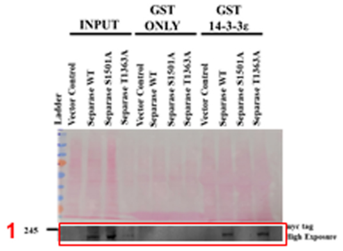

Entire blot

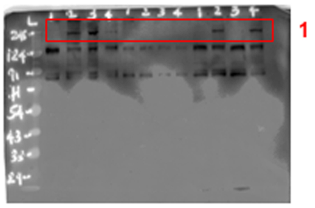

Note: Individually developed blots were used in the manuscript.

Journal of Cell Science • Supplementary information

BLOT TRANSPARENCY 10

Image in the manuscript

Supplementary 7A

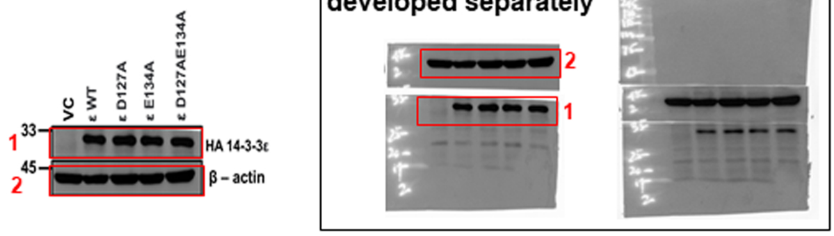

Image in the manuscript

Supplementary 7D

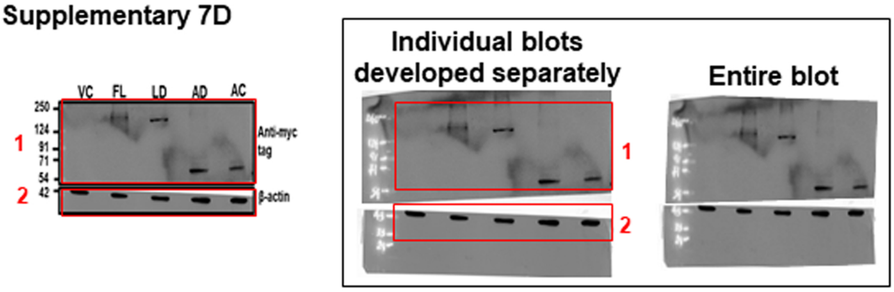

Image in the manuscript

Supplementary 7F

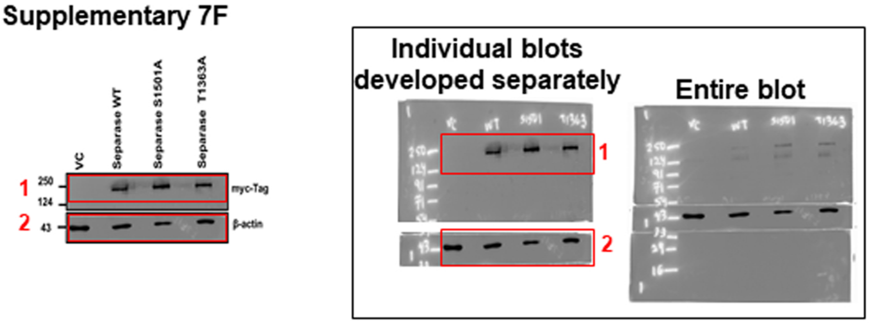

Note: Individually developed blots were used in the manuscript.

Fig. S8. Images of Western blots used for the manuscript.

Journal of Cell Science • Supplementary information

Table S1. List of chemicals and suppliers

Chemicals, Enzymes and other reagents	Company	Catalog number
Polyethylenimine (PEI)	Polyscience, Inc.	23966
Lipofectamine 3000	Invitrogen	L3000-008
Albumin bovine fraction V (BSA)	Sigma	A-9418
Protease inhibitors	Sigma	P-8340
Phosphatase inhibitors	Sigma	P5726
Protein G Sepharose beads	Amersham	17-0618-01
Glutathione Sepharose beads	Cytiva	17075601
Nitrocellulose membrane	MDI	SCNX8401XXXX101
Clarity™ Western ECL	BioRad	170-5061
Femto Western ECL	Gbiosciences	786003
Ammonium persulfate	SRL	84569
TEMED	Sigma	T7024MKBW9634V
DAPI	Sigma, Ibidi	D-9542, Cat #50011
IPTG	Sigma	I-5502
Sepin1 (10µM)	Med Chem Express,	HY-117522
BI2536 (200nM)	Med Chem Express	HY-50698
Mimosine (200µM)	Sigma	M0253
Nocodazole (100nM)	Sigma	M-1404
RO3306 (10µM)	Sigma	SML-0569
DMEM	Invitrogen	12800-017
F12 nutrient media	Invitrogen	21700-075
Fetal Bovine Serum	Invitrogen	10270-106
Tween 20	Sigma	P1379
Protein molecular weight markers	Puregene and SRL	PG-PMT 2922 and MBT092-10N
G418	Sigma	A1720
Puromycin	Sigma	P-8833
Duolink® In Situ Red Starter Kit Mouse/Rabbit (PLA Kit)	Sigma	DUO92101
Reduced Glutathione	Sigma	G-6013

Journal of Cell Science • Supplementary information

Table S2. List of oligonucleotides.

Gene targeted or amplified	Forward Sequence	Reverse Sequence
Separase Large Domain	GACCTCGAGAGGAGCTTCAA AAGAGTCAACTTTGGG	GCGGCCGCTCACCTGAGGAT CTAAAGCTC
Separase Activation Domain	CAGTCACTCGAGCTGGATTC CAGCAAGAAGAAGC	GTCACTGCGGCCGCTCACCG CAGAGAGACAGGCAAGC
Separase Autocatalytic Domain	GTCACTCTCGAGTCCCATTCA CCCACCTGTGAC	CTGACAGCGGCCGCCTATTAA AGTGATCCTCGGTGCTTCTGG
Separase S1501A	GACTGACAACTGGAGAAAAA TGGCCTTCGAAATCCTCAGG GGCTCTGACGGGG	CCCCGTCAGAGCCCCTGAGG ATTTCGAAGGCCATTTTTCTCC AGTTGTCAGTC
Separase T1363A	GCTGGCCCTCATGTCCCCTT CGCCGTGTTCGAAGAAGTCT GCCCTACAGAG	CTCTGTAGGGCAGACTTCTTC GAACACGGCGAAGGGGACAT GAGGGCCAGC
PLK1 S99D	CGCACCAGAGGGAGAAGATG GATATGGAAATATCGATTCAC CGCAGCCTCGC	GCGAGGCTGCGGTGAATCGA TATTTCCATATCCATCTTCTCC CTCTGGTGCG
PLK1 S99E	CGCACCAGAGGGAGAAGATG GAAATGGAAATATCGATTCAC CGCAGCCTCGC	GCGAGGCTGCGGTGAATCGA TATTTCCATTTCCATCTTCTCC CTCTGGTGCG
PLK1 S99A	AAGCCGCACCAGAGGGAGAA GATGGCAATGGAAATATCCAT TCACCGCAGC	GCTGCGGTGAATGGATATTTC CATTGCCATCTTCTCCCTCTG GTGCGGCTT
PLK1 T210A	GAATATGACGGGGAGAGGAA GAAGGCCTTGTGTGGGACTC CTAATTACATAGC	GCTATGTAATTAGGAGTCCCA CACAAGGCCTTCTTCCTCTCC CCGTCATATTC
14-3-3ε E134A	ATGAAAGGGGACTACCACAG GTACCTGGCAGCATTTGCCA CAGGAAAC	GTTTCCTGTGGCAAATGCTGC CAGGTACCTGTGGTAGTCCCC TTTCAT
14-3-3ε D127AE134A	ATGAAAGGGGCCTACCACAG GTACCTGGCAGCATTTGCCA CAGGAAAC	GTTTCCTGTGGCAAATGCTGC CAGGTACCTGTGGTAGGCCC CTTTCAT
14-3-3ε BamHI and 14-3-3ε XhoI	AGGATCCATGGATGATCGAG AGG	ACTCGAGTCACTGATTTTCGT C
14-3-3ε gRNA1	CACCGTCGATCATCCATAGC GGCAG	AAACCTGCCGCTATGGATGAT CGAC
14-3-3ε gRNA2	CACCGATCCATAGCGGCAGC GGCTC	AAACGAGCCGCTGCCGCTAT GGATC
14-3-3 ζ gRNA	CACCGTGTGACTGATCGACA ATCCC	AAACGGGATTGTCGATCAGTC ACAC
14-3-3ζ gRNA2	CACCGAGATATCTGCAATGAT GTAC	AAACGTACATCATTGCAGATA TCTC

Journal of Cell Science • Supplementary information

Table S3. Antibodies and dilutions for Western blot and immunofluorescence.

Antibodies	Company	Catalog number
14-3-3ε (T-16) (Rabbit, 1:1000)	Santa Cruz	SC-1020
14-3-3ε (F-3) (Mouse, 1:1000)	Santa Cruz	393177
HA (Mouse, 1:3000)	Sigma	11583816001
β-actin (Mouse, 1:2000)	Sigma	A-5316
Plk1 (Rabbit, 1:2500)	CST/ILS	4513
Separase (Rabbit, 1:1000)	Abcam	Ab-3762
Myc (Rabbit, 1:1000)	CST	2278T
Myc (Mouse, 1:1000)	CST	2276S
Pericentrin (Rabbit 1:500)	Abcam	Ab448
Cep68 (Rabbit 1:200)	Abcam	Ab-91455
GT335 (Mouse 1:100)	Adipogen	AG-20B-0020-C100
Centrin2 (Rabbit 1:200)	Santa Cruz	SC-27793
α-tubulin (Rabbit 1:75)	Abcam	Ab-52866
Cep215 (Rabbit 1:200)	Sigma	06-1398
Rootletin (Rabbit 1:100)	Novus	NBPI-80820
ODF2 (Mouse1:50)	Novus	H0004957-M01
cdc25C (Rabbit 1:50)	Santa Cruz	SC-13138
SAS6 (Mouse 1:50)	Santa Cruz	SC-81431
CEP110 (Rabbit 1:100)	Cloud Clone	CAB870Hu01
γ-tubulin (Mouse 1:100)	Santa Cruz	SC-17787
phospo-PLK1 T210 (D5H7) (Rabbit 1:50)	CST	9062
HRP Secondary Antibody (anti Mouse, 1:2500)	Pierce	32430
HRP Secondary Antibody (anti Rabbit, 1:5000)	Pierce/Thermo Fisher	31460
Alexa Fluor 488 anti-mouse (1:200)	Invitrogen	A-11001
Alexa Fluor 488 anti-rabbit (1:200)	Invitrogen	A-11008
Alexa Fluor 568 anti-mouse (1:200)	Invitrogen	A-11004
Alexa Fluor 568 anti-rabbit (1:200)	Invitrogen	A-11011
Alexa Fluor 633 anti-rabbit (1:200)	Invitrogen	A-21072
Alexa Fluor 647 anti-rabbit (1:200)	Invitrogen	A-31573

Journal of Cell Science • Supplementary information

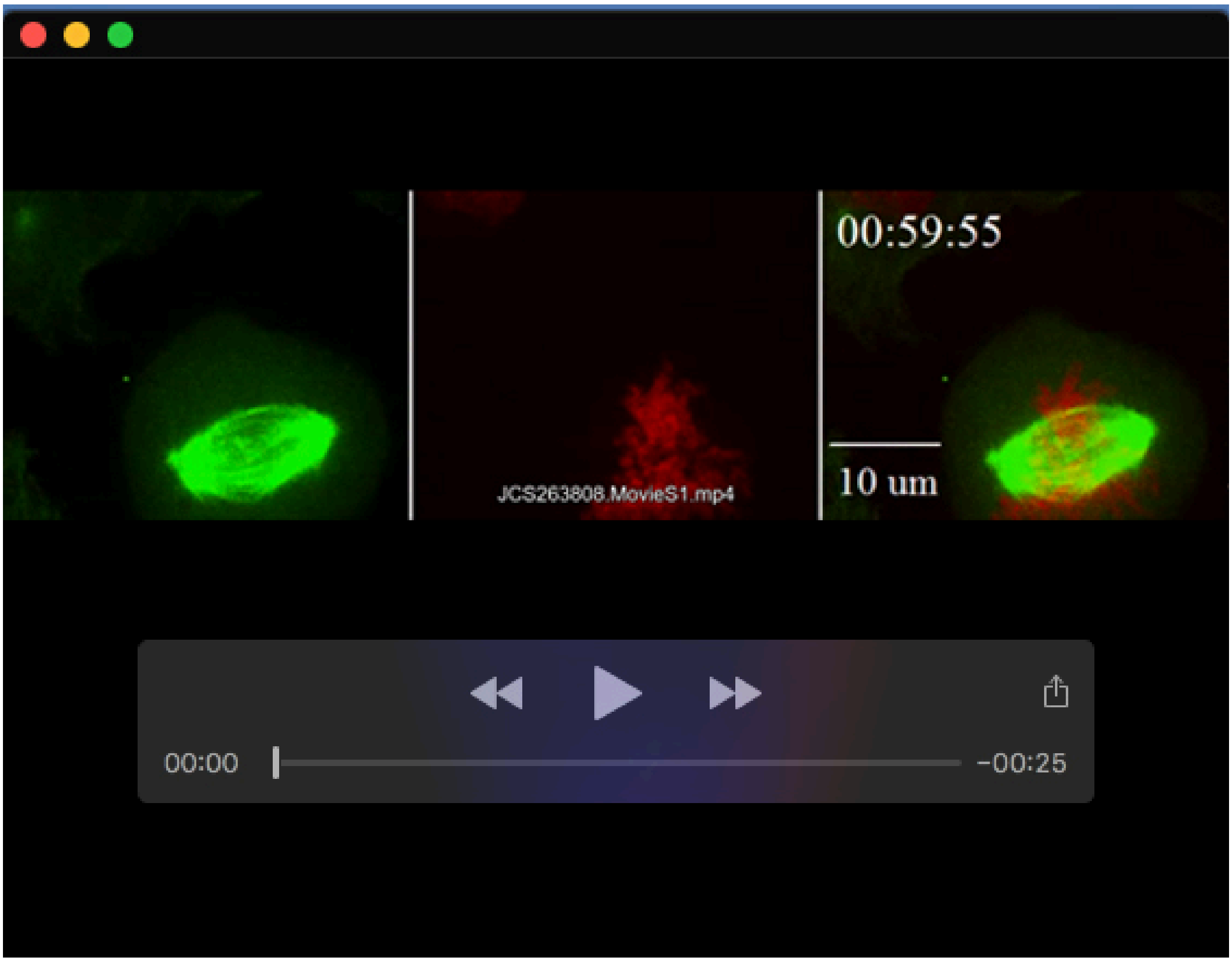

Movie 1. Mitotic phenotypes observed upon expression of WT and mutant 14-3-3ε. HeLa Kyoto cells stably expressing the vector control were imaged over time on an Olympus 3i spinning disc microscope at intervals of 20 minutes over 20 hours. The time stamp is on the top right, and the scale bar is on the bottom left. Scale bar = 10μm.

Journal of Cell Science • Supplementary information

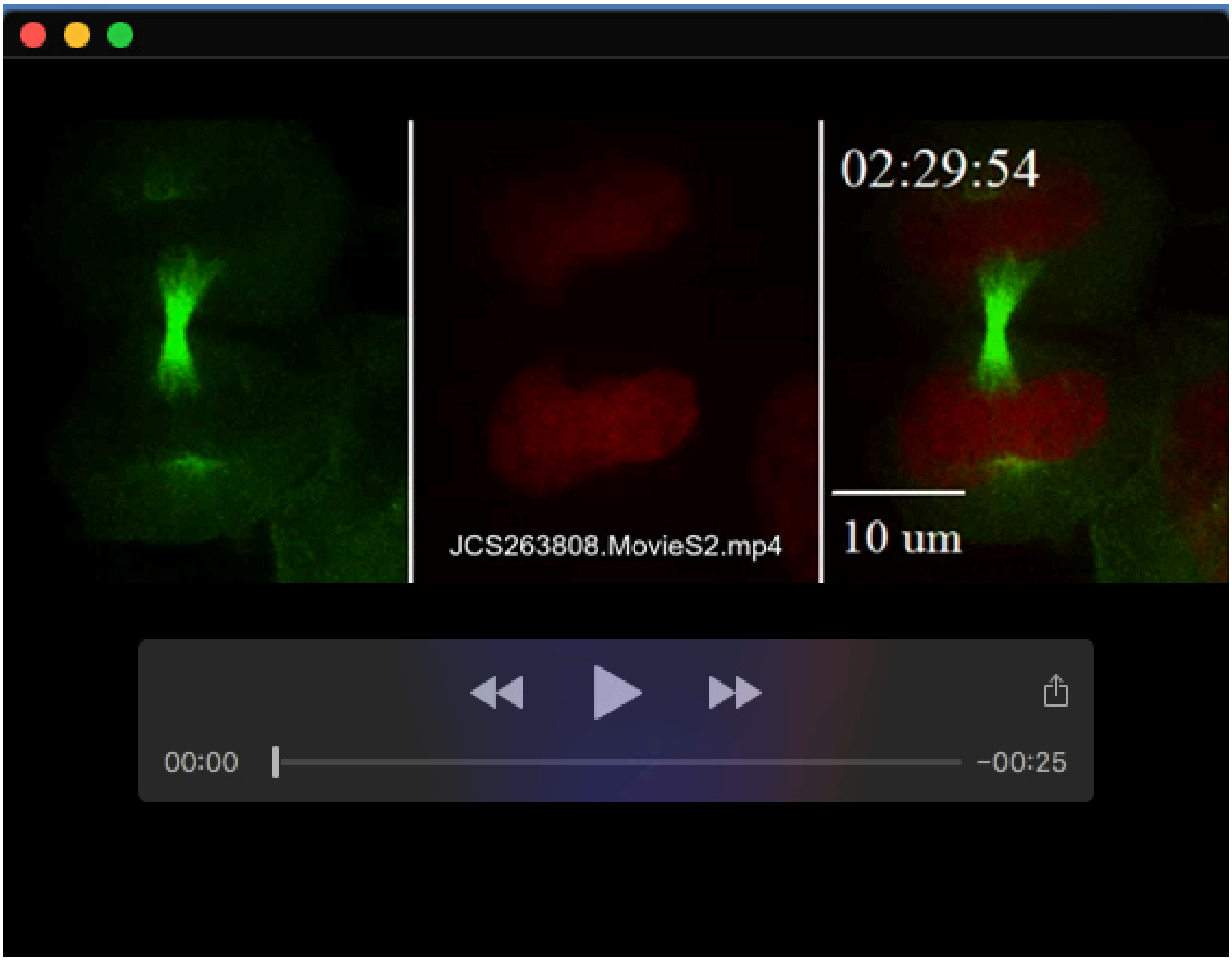

Movie 2. Mitotic phenotypes observed upon expression of WT and mutant 14-3-3ε. WT 14-3-3ε were imaged over time on an Olympus 3i spinning disc microscope at intervals of 20 minutes over 20 hours. The time stamp is on the top right, and the scale bar is on the bottom left. Scale bar = 10µm.

Journal of Cell Science • Supplementary information

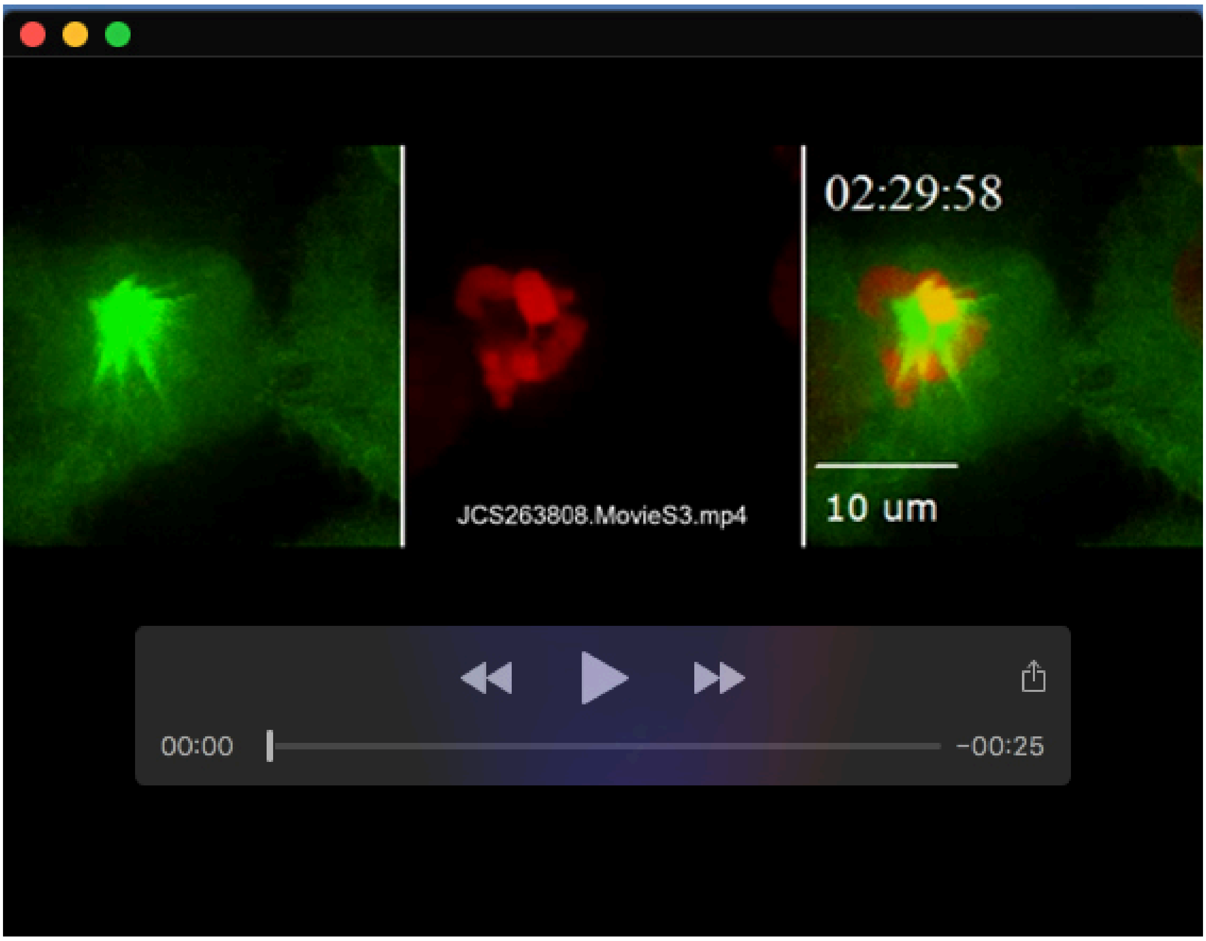

Movie 3. Mitotic phenotypes observed upon expression of WT and mutant 14-3-3ε. The 14-3-3ε mutants D127A were imaged over time on an Olympus 3i spinning disc microscope at intervals of 20 minutes over 20 hours. The time stamp is on the top right, and the scale bar is on the bottom left. Scale bar = 10μm.

Journal of Cell Science • Supplementary information

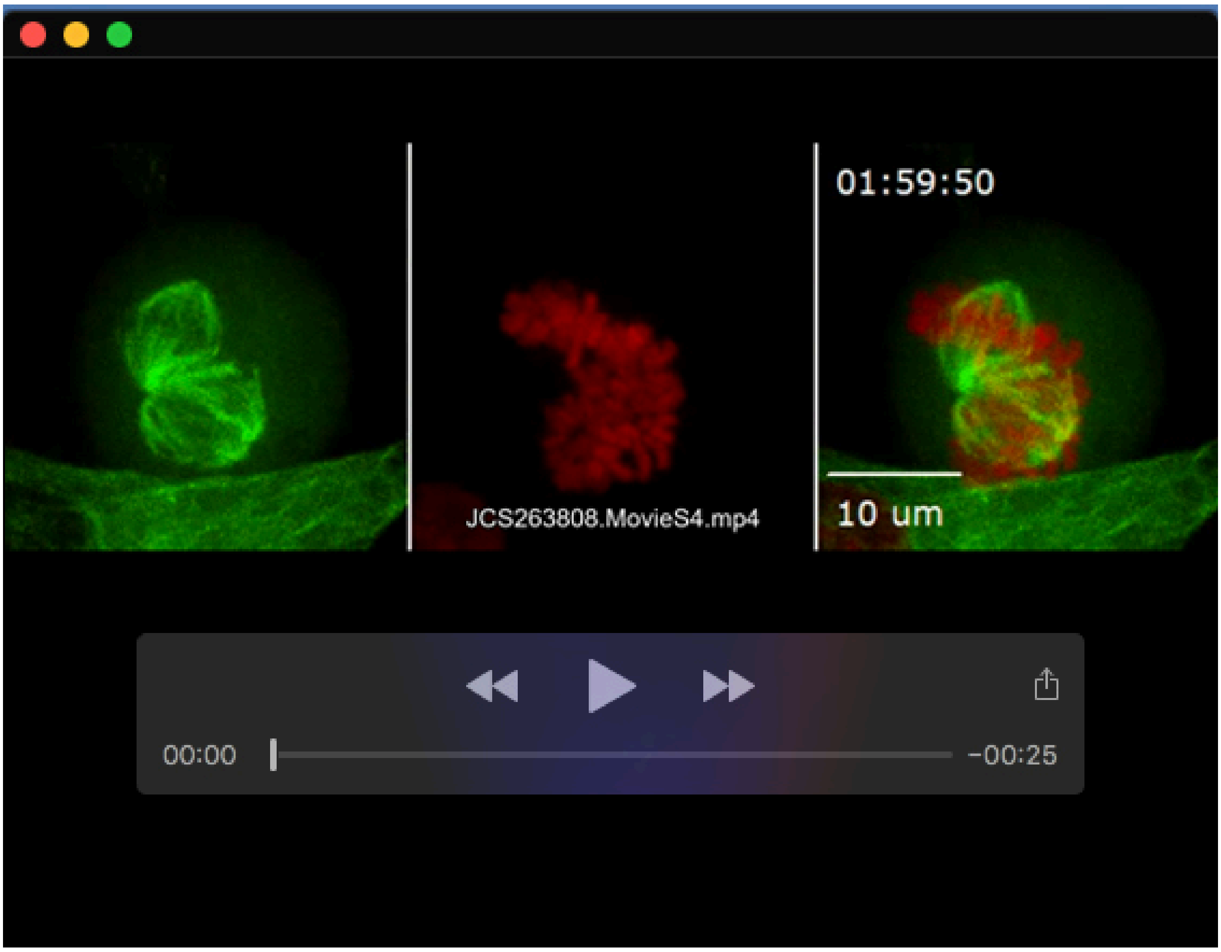

Movie 4. Mitotic phenotypes observed upon expression of WT and mutant 14-3-3ε. E134A were imaged over time on an Olympus 3i spinning disc microscope at intervals of 20 minutes over 20 hours. The time stamp is on the top right, and the scale bar is on the bottom left. Scale bar = 10μm.

Journal of Cell Science • Supplementary information

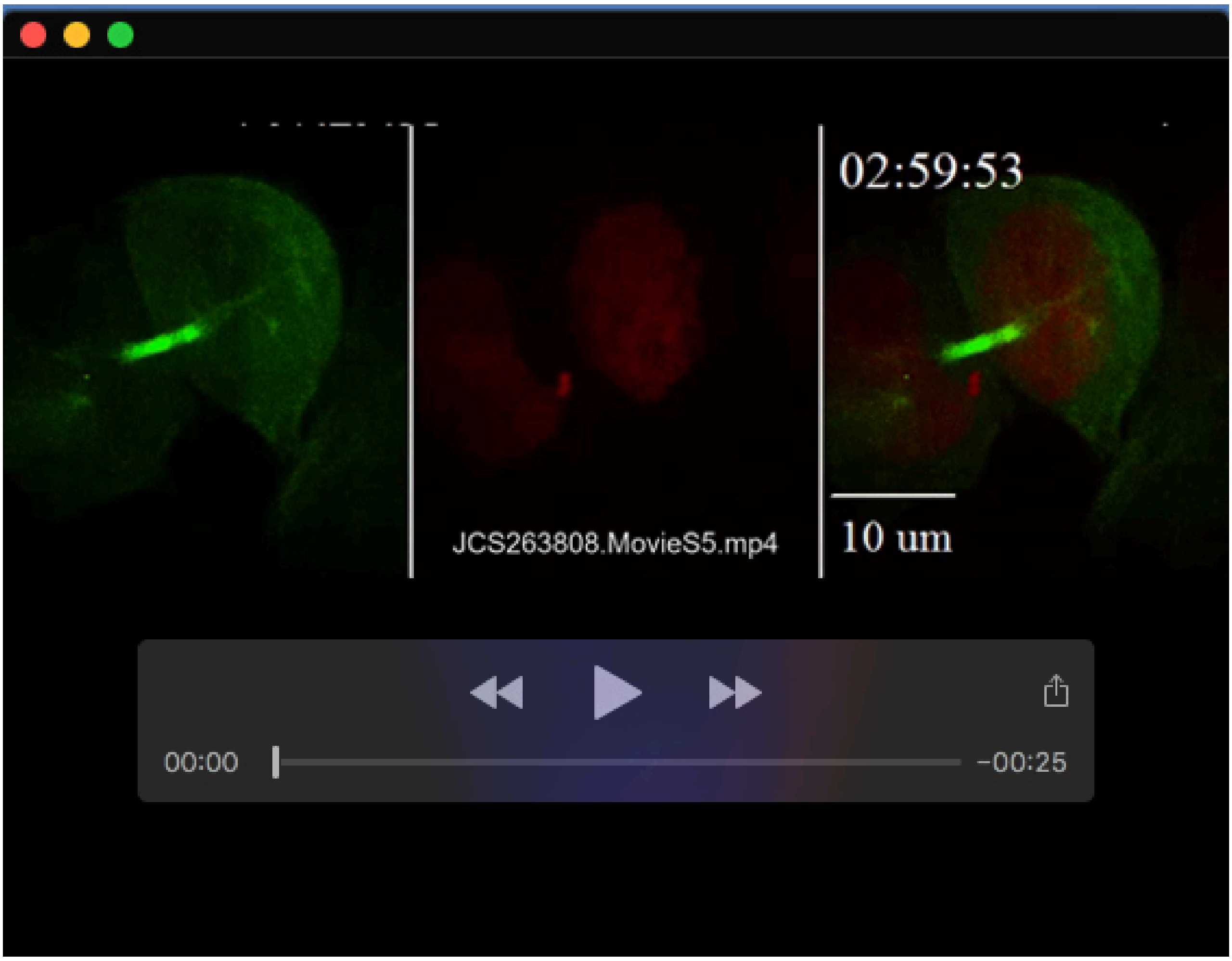

Movie 5. Mitotic phenotypes observed upon treatment of cells expressing WT and mutant 14-3-3ε with Plk1 and Separase inhibitors. HeLa Kyoto cells stably expressing WT 14-3-3ε, were treated with the vehicle control. The cells were imaged over time on an Olympus 3i spinning disc microscope at intervals of 20 minutes over 20 hours. The time stamp is on the top right, and the scale bar is on the bottom left. Scale bar = 10μm.

Journal of Cell Science • Supplementary information

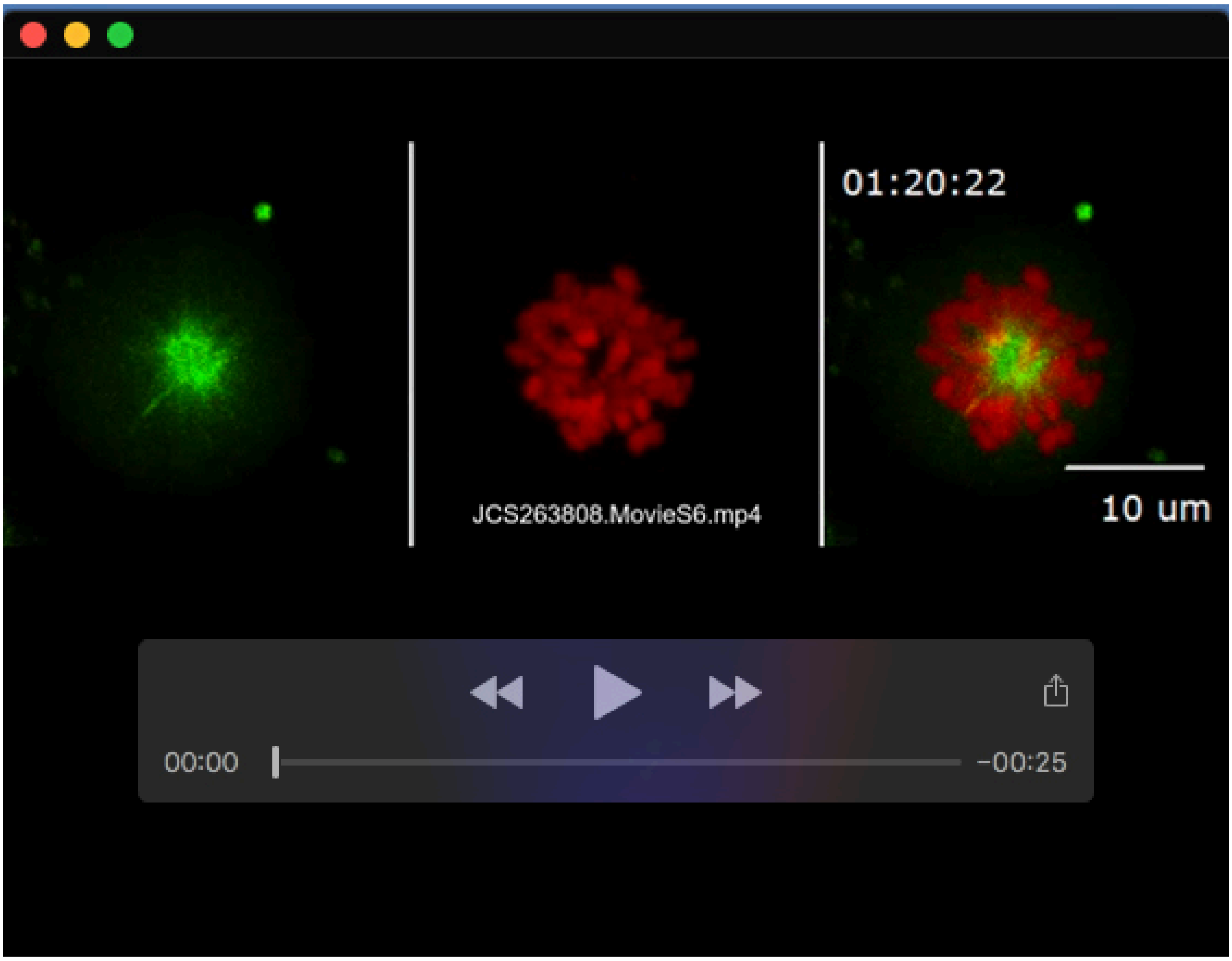

Movie 6. Mitotic phenotypes observed upon treatment of cells expressing WT and mutant 14-3-3ε with Plk1 and Separase inhibitors. HeLa Kyoto cells stably expressing WT 14-3-3ε, the Plk1 inhibitor BI2536. The cells were imaged over time on an Olympus 3i spinning disc microscope at intervals of 20 minutes over 20 hours. The time stamp is on the top right, and the scale bar is on the bottom left. Scale bar = 10μm.

Journal of Cell Science • Supplementary information

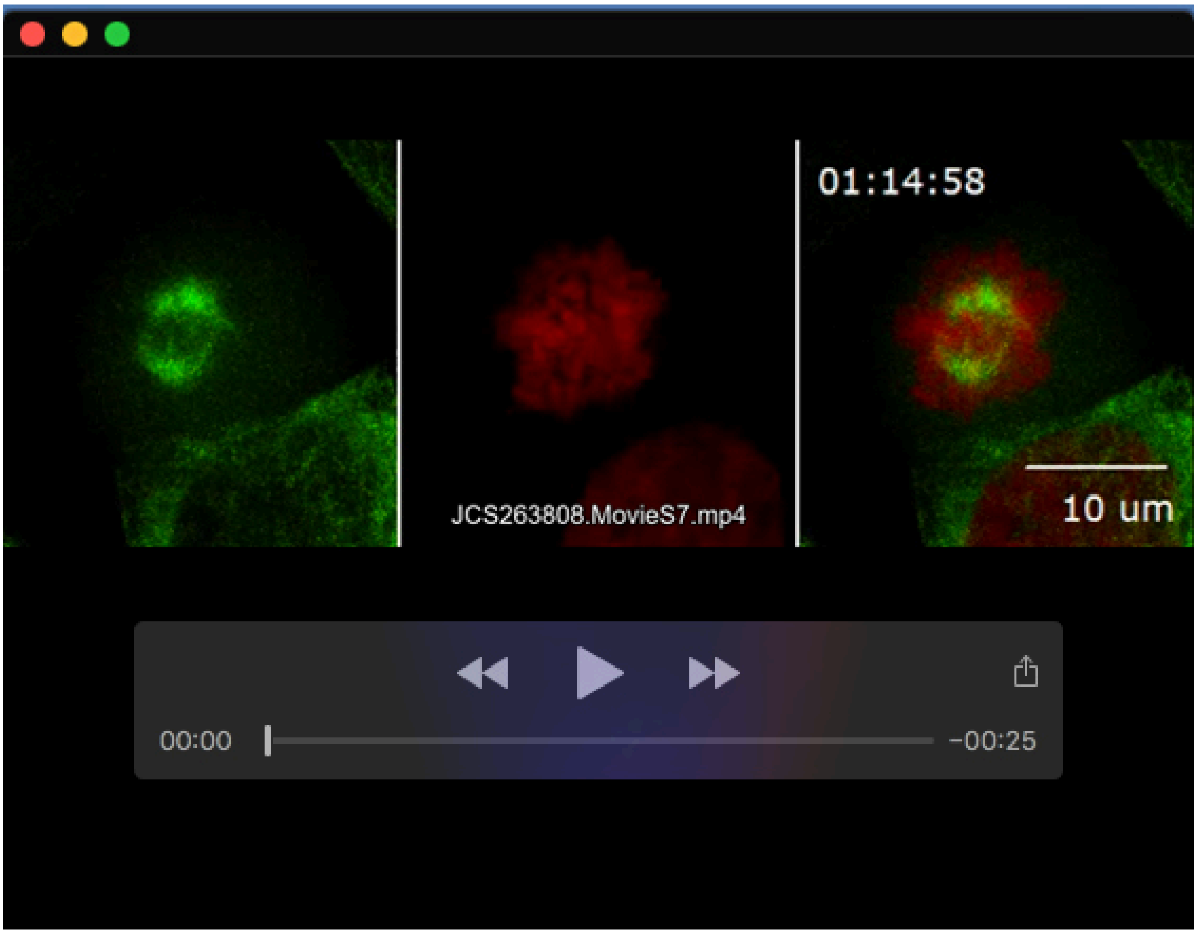

Movie 7. Mitotic phenotypes observed upon treatment of cells expressing WT and mutant 14-3-3ε with Plk1 and Separase inhibitors. HeLa Kyoto cells stably expressing WT 14-3-3ε, or both inhibitors. The cells were imaged over time on an Olympus 3i spinning disc microscope at intervals of 20 minutes over 20 hours. The time stamp is on the top right, and the scale bar is on the bottom left. Scale bar = 10μm.

Journal of Cell Science • Supplementary information

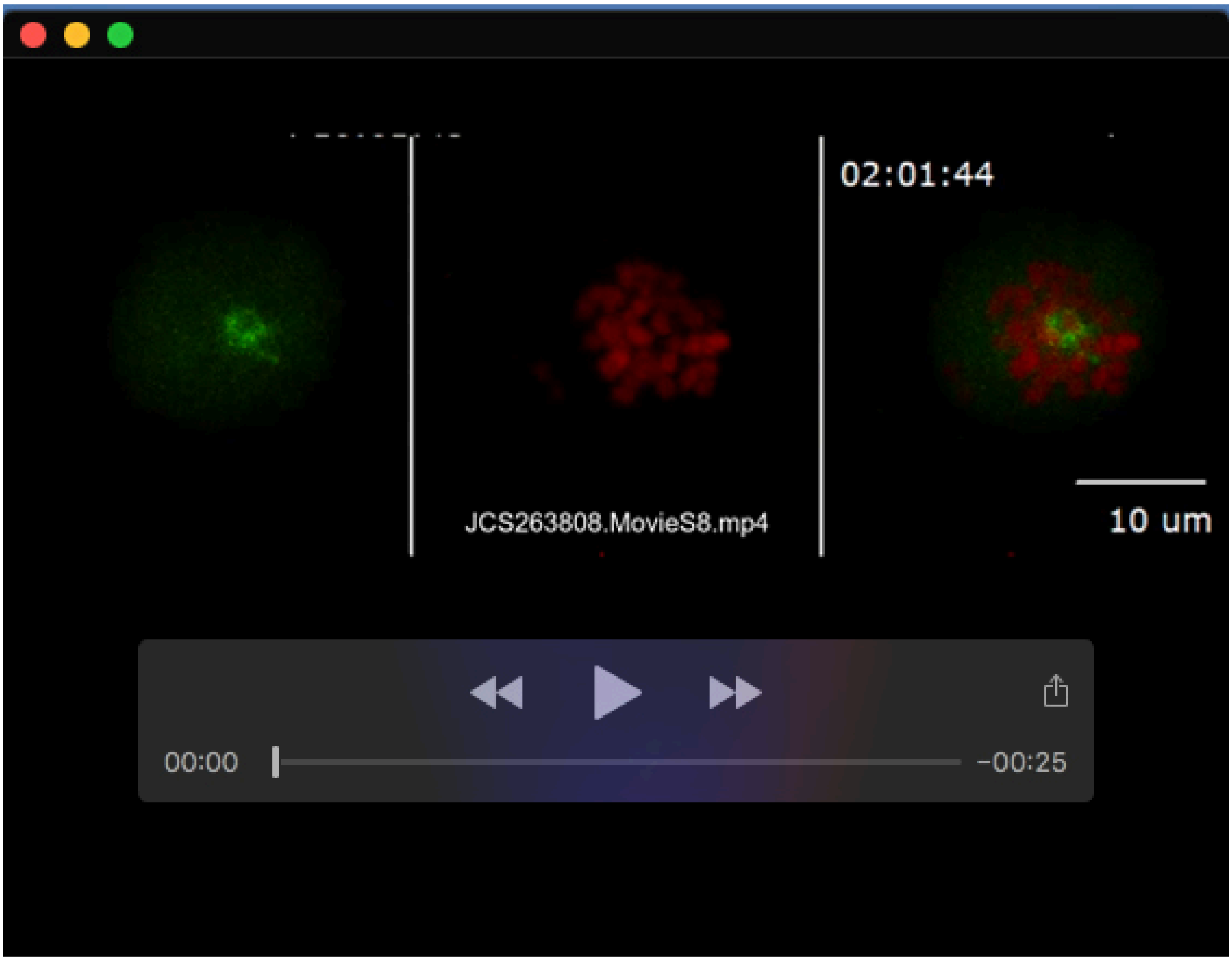

Movie . Mitotic phenotypes observed upon treatment of cells expressing WT and mutant 14-3-3ε with Plk1 and Separase inhibitors. HeLa Kyoto cells stably expressing WT 14-3-3ε, or both inhibitors. The cells were imaged over time on an Olympus 3i spinning disc microscope at intervals of 20 minutes over 20 hours. The time stamp is on the top right, and the scale bar is on the bottom left. Scale bar = 10µm.

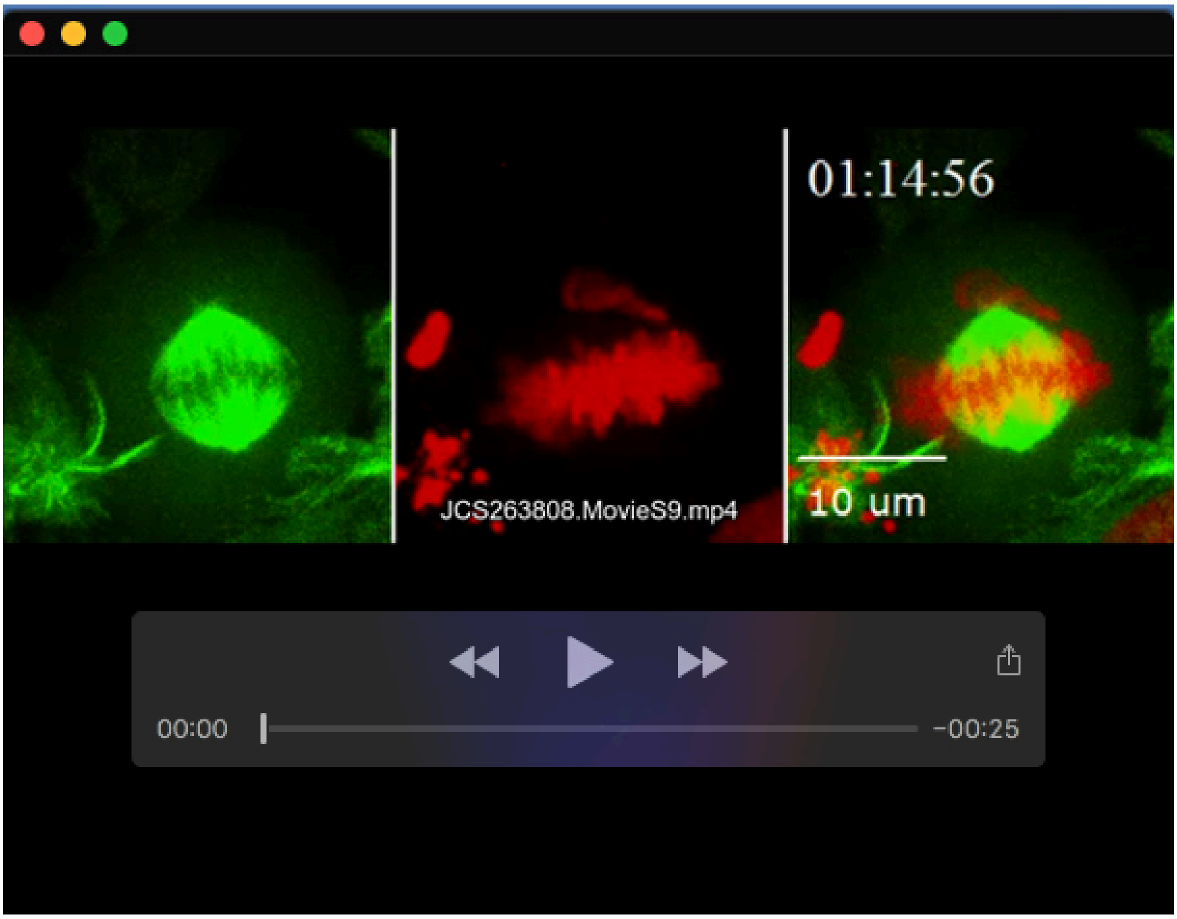

Movie . Mitotic phenotypes observed upon treatment of cells expressing WT and mutant 14-3-3ε with Plk1 and Separase inhibitors. The 14-3-3ε E134A mutant were treated with the vehicle control. The cells were imaged over time on an Olympus 3i spinning disc microscope at intervals of 20 minutes over 20 hours. The time stamp is on the top right, and the scale bar is on the bottom left. Scale bar = 10µm.

Journal of Cell Science • Supplementary information

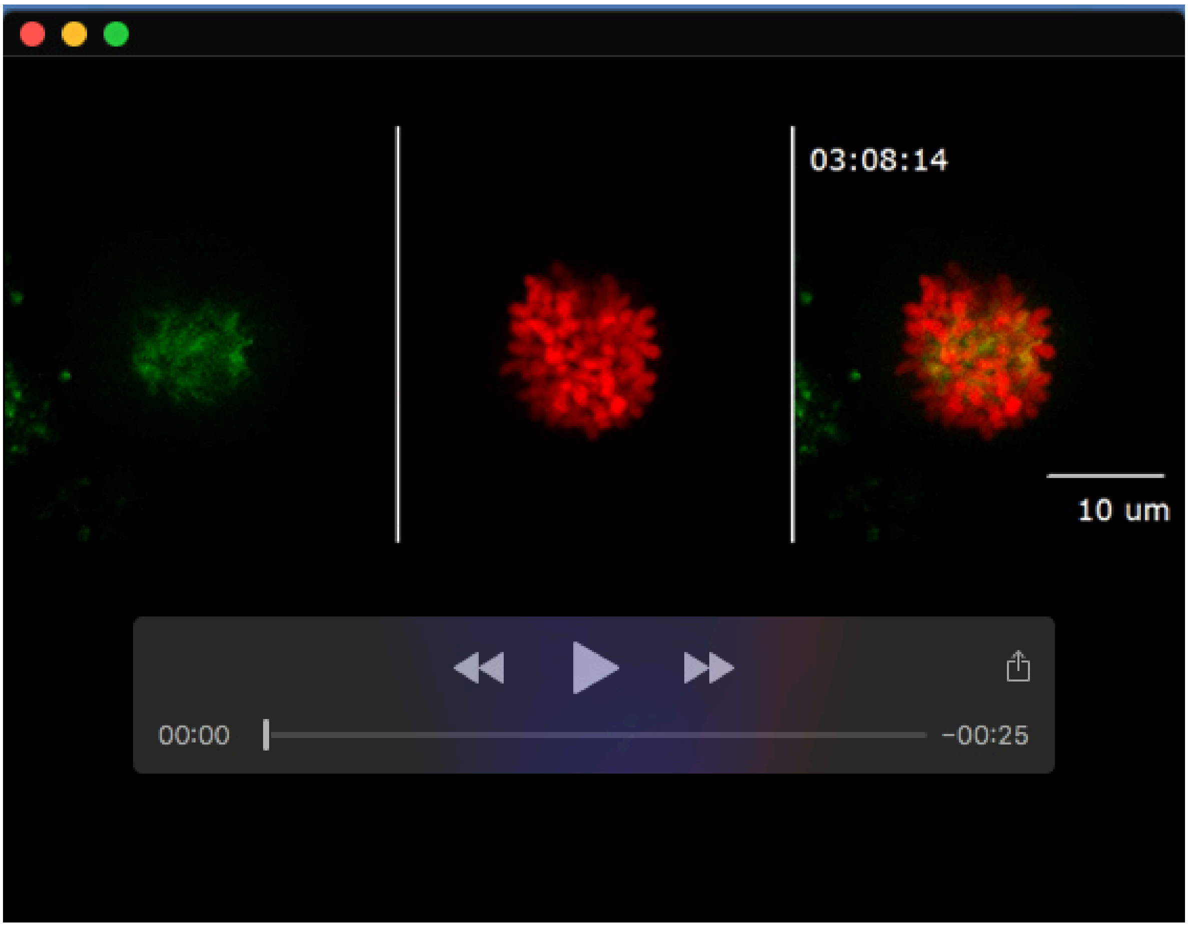

Movie 1 . Mitotic phenotypes observed upon treatment of cells expressing WT and mutant 14-3-3ε with Plk1 and Separase inhibitors. The 14-3-3ε E134A mutant were treated with the vehicle control, the Plk1 inhibitor BI2536. The cells were imaged over time on an Olympus 3i spinning disc microscope at intervals of 20 minutes over 20 hours. The time stamp is on the top right, and the scale bar is on the bottom left. Scale bar = 10µm.

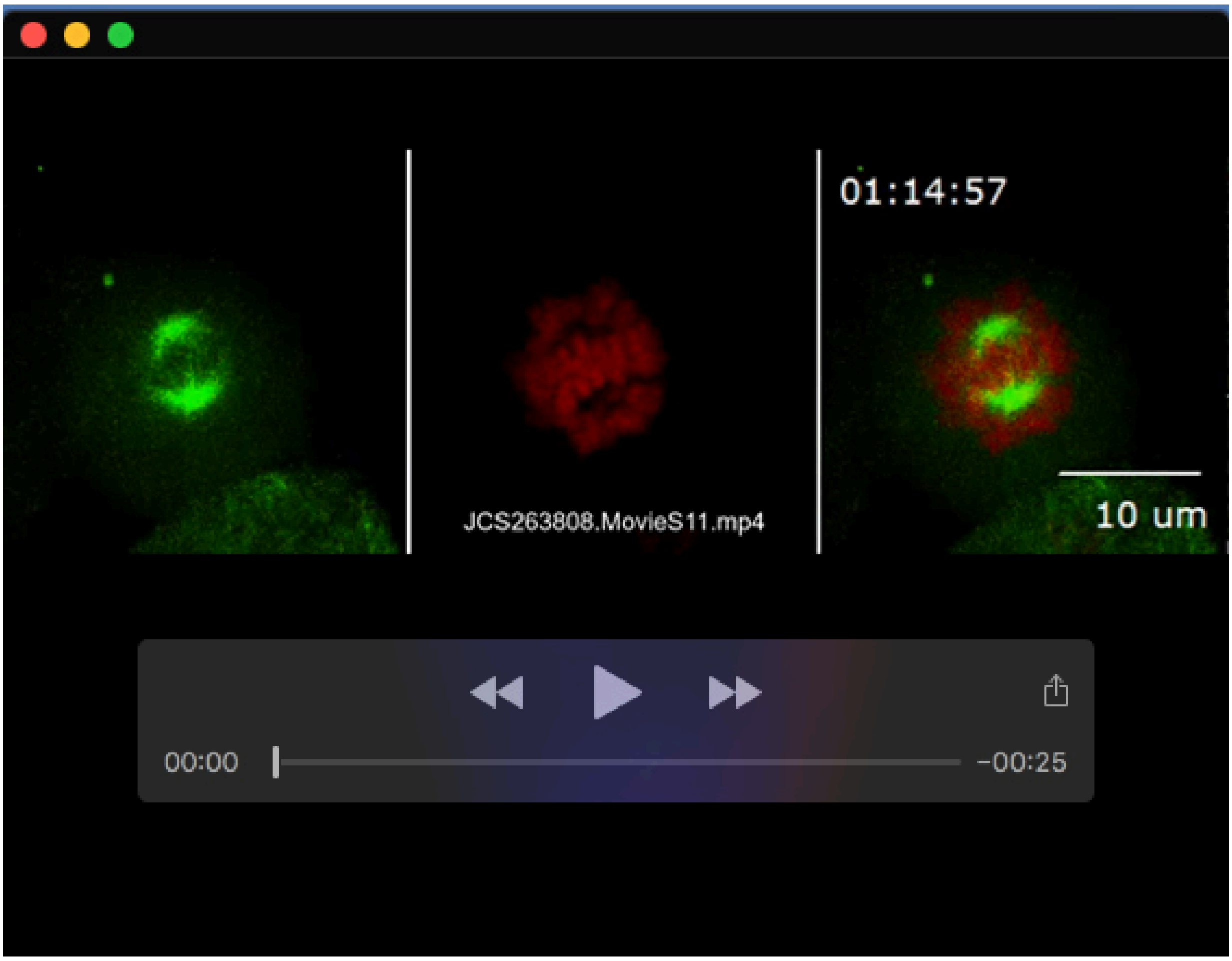

Movie 11. Mitotic phenotypes observed upon treatment of cells expressing WT and mutant 14-3-3ε with Plk1 and Separase inhibitors. The 14-3-3ε E134A mutant, the Separase inhibitor Sepin-1. The cells were imaged over time on an Olympus 3i spinning disc microscope at intervals of 20 minutes over 20 hours. The time stamp is on the top right, and the scale bar is on the bottom left. Scale bar = 10μm.

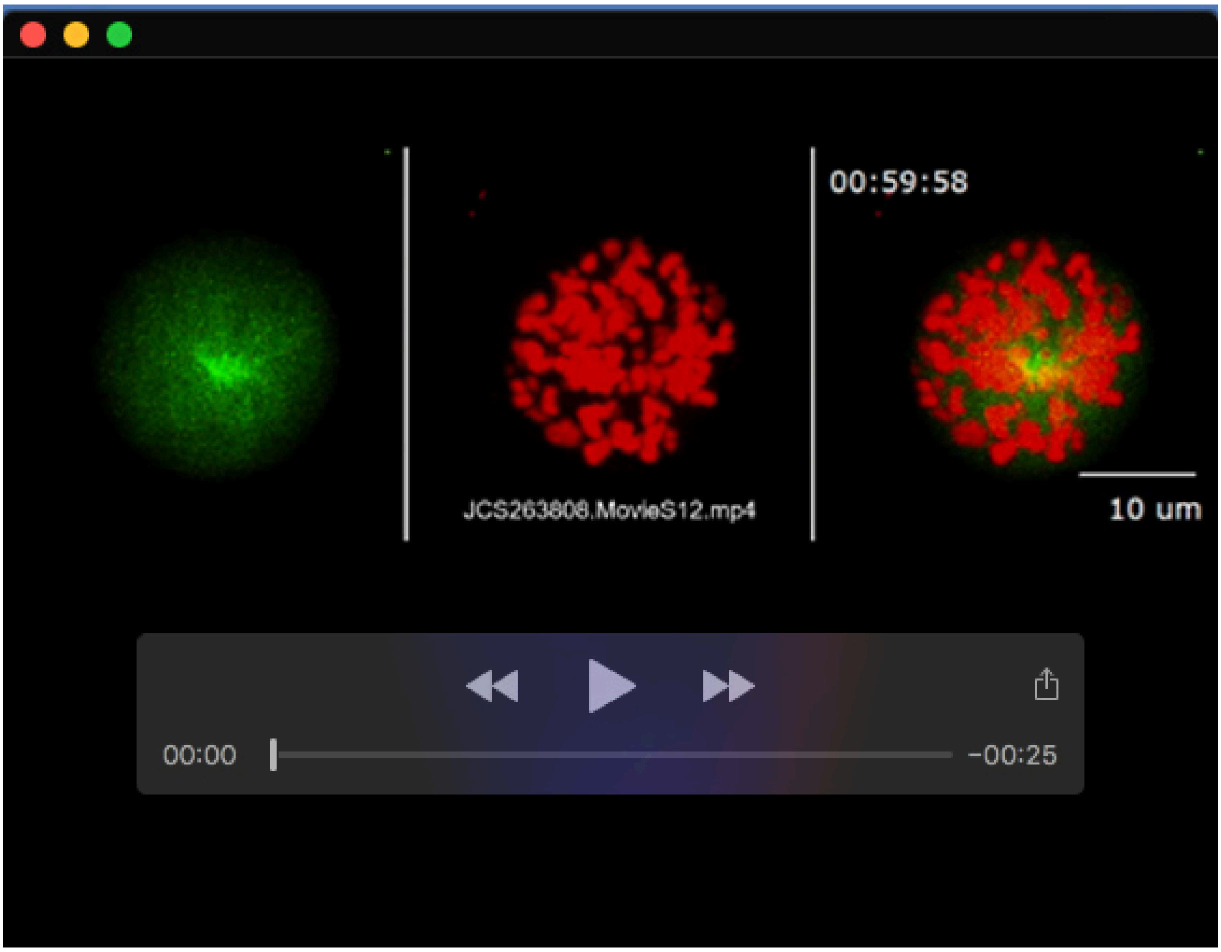

Movie 12. Mitotic phenotypes observed upon treatment of cells expressing WT and mutant 14-3-3ε with Plk1 and Separase inhibitors. The 14-3-3ε E134A mutant or both inhibitors. The cells were imaged over time on an Olympus 3i spinning disc microscope at intervals of 20 minutes over 20 hours. The time stamp is on the top right, and the scale bar is on the bottom left. Scale bar = 10µm.
